# A lineage-specific protein network at the trypanosome nuclear envelope

**DOI:** 10.1080/19491034.2024.2310452

**Published:** 2024-04-11

**Authors:** Erin R. Butterfield, Samson O. Obado, Simon R. Scutts, Wenzhu Zhang, Brian T. Chait, Michael P. Rout, Mark C. Field

**Affiliations:** a School of Life Sciences, University of Dundee, Dundee, UK; b Laboratory of Cellular and Structural Biology, The Rockefeller University, New York, NY, USA; c Department of Pathology, University of Cambridge, Cambridge, UK; d Laboratory of Mass Spectrometry and Gaseous Ion Chemistry, The Rockefeller University, New York, NY, USA; e Biology Centre, Czech Academy of Sciences, Institute of Parasitology, České Budějovice, Czech Republic

**Keywords:** Nucleus, nuclear pore complex, nuclear lamina, molecular evolution, comparative genomics, AlphaFold

## Abstract

The nuclear envelope (NE) separates translation and transcription and is the location of multiple functions, including chromatin organization and nucleocytoplasmic transport. The molecular basis for many of these functions have diverged between eukaryotic lineages. *Trypanosoma brucei*, a member of the early branching eukaryotic lineage Discoba, highlights many of these, including a distinct lamina and kinetochore composition. Here, we describe a cohort of proteins interacting with both the lamina and NPC, which we term lamina-associated proteins (LAPs). LAPs represent a diverse group of proteins, including two candidate NPC-anchoring pore membrane proteins (POMs) with architecture conserved with *S. cerevisiae* and *H. sapiens*, and additional peripheral components of the NPC. While many of the LAPs are Kinetoplastid specific, we also identified broadly conserved proteins, indicating an amalgam of divergence and conservation within the trypanosome NE proteome, highlighting the diversity of nuclear biology across the eukaryotes, increasing our understanding of eukaryotic and NPC evolution.

## Introduction

The nucleus is delineated by the nuclear envelope (NE) and structurally supported by an internal lamina that constitutes a nucleoskeleton, various lamina-interacting proteins and the nuclear pore complex (NPC). The lamina mediates chromatin organization, gene regulation, maintenance of nuclear integrity and localization of the NPCs [1]. In some organisms, the NE disassembles during mitosis, while in others remains essentially intact, with no known correlation between lamina composition and open or closed mitosis [2,3]. In metazoa, the lamina is composed of ~60 kDa lamins, type V intermediate filament proteins. Lamin orthologs are widely distributed across eukaryotes, and it is likely that the last eukaryotic common ancestor (LECA) possessed a lamin-based lamina [3]. However, lamins are not universal and many lineages lack lamin genes, including *Saccharomyces cerevisiae* and other fungi, plants and many protists [4,5]. In some lineages, lamins have been replaced by protein analogs retaining highly equivalent functions [4,6–9], suggesting that structural and functional demands can be met by alternate mechanisms.

In many organisms, lamina-interacting proteins include the LINC (Linker of Nucleoskeleton and Cytoskeleton) complex, which connects the nuclear lamina and cytoskeleton and functions in nuclear positioning [10], and LEM-domain (LAP2, emerin, MAN1) proteins involved in chromosome tethering and NE repair through ESCRT recruitment [11,12]. Mutations in lamins or lamin-associated genes can give rise to laminopathies, many of which are associated with alterations in chromosome stability and gene expression and result in severe developmental disorders [11,13]. The NPC, a multiprotein complex that supports all known transport into and out of the nucleus, also interacts with the lamins. The NPC is composed of a core scaffold of nucleoporins (or Nups) with a combination of β-propeller/α-solenoid domain architecture [14], that anchor FG-Nups, proteins having extensive disordered domains containing Phe-Gly repeats. FG-Nups occupy the NPC central channel and mediate selective gating [15]. The nuclear face of the NPC, and the associated nuclear basket, interacts with mRNA processing complexes and chromatin to modulate gene expression [14,16]. Significantly, the NPC is subject to evolutionary sculpting, with distinct arrangements of subdomains in different taxa [17,18].

*Trypanosoma brucei* is a unicellular parasite causing human African trypanosomiasis and Nagana in animals [19]. While currently no longer a major public health threat, *T. brucei* remains an important model for evolutionary cell biology, due to early divergence from the main eukaryote lineage and ease of manipulation [20–22]. Multiple nuclear functions are represented in *T. brucei* by very divergent systems, including mRNA processing and splicing, chromosome segregation, heterochromatinization and monoallelic exclusion at telomeric expression sites [4,23]. Divergence within the nuclear proteome of trypanosomes extends to histones, resulting in altered nucleosome structures [24] and elements of the transcriptional system [25], reflecting an extremely deep divide between *T. brucei* and animals, fungi, plants and most protists. Furthermore, many proteins identified at the nuclear envelopes of mammalian cells, trypanosomes and other organisms are lineage-specific; for example, animals do not share the majority of nuclear envelope *trans*-membrane (NET) proteins with even ‘closely’ related taxa such as yeasts [26].

Two lamina components, NUP-1 and NUP-2, have been identified in *T. brucei* and have major roles in nuclear organization and heterochromatin silencing [8,9]. NUP-1 is a 407 kDa coiled-coil repetitive protein required for maintenance of nuclear integrity, maintaining NPC position, chromosome organization and antigenic variation [8]. NUP-2 is 170 kDa and also has coiled-coil architecture but lacks a repetitive structure. Similar to NUP-1, NUP-2 is also required for maintenance of nuclear structure, chromosome organization and antigenic variation; significantly, both NUP-1 and NUP-2 are codependent for correct localization, indicating intimate functional – and likely physical – contacts [9]. NUP-1 and NUP-2 are present across the kinetoplastids, but not beyond, and, for example, they are absent from *Euglena gracilis* [8,9,27].

The trypanosome NPC has been characterized by comparative genomics and proteomics [28–30]. No proteins or genes resembling the LINC complex or LEM-domain components have been identified either *in silico* or through extensive proteomics [3,4,9,28,29], suggesting that either the trypanosome NE truly has highly distinct composition or that many protein sequences are too divergent for identification. To expand our understanding of the NE, and in particular connections with the lamina and NPC, we have identified trypanosome NE proteins through direct, unbiased proteomics. Remarkably, most of these proteins are specific to the trypanosome lineage, supporting a paradigm of distinct NE composition between lineages. However, we identify a structural homolog of the NPC membrane anchoring proteins Pom152 and GP210, suggesting a conserved mechanism for NPC interaction with the membrane.

## Methods

### Structural annotation and prediction

Coiled-coil domains were predicted with COILS [31] using weighted and unweighted scans with a sliding window of 28 residues. Signal peptides and *trans*-membrane domains were predicted using Phobius [32,33], SignalP v3.0 or v4.1 [34–36] and TMHMM (http://www.cbs.dtu.dk/services/TMHMM/). NCBI conserved domain search [37], HHPred [38,39], Prosite [40,41] and HELIQUEST [42] were used to predict domains and motifs. For HHPred [38,39], sequences were searched against the PDB70 [43] and PFAM-A [44] databases. Hits (models and protein families) returned were considered homologous if they had a probability >95%. Hits with >50% probability were considered likely if multiple hits contained the same domain or if the domain was identified through two or more methods. cNLS Mapper [45], NucPred [46] and NLStradamus [47] were used to predict nuclear localization and identify nuclear localization signals. Precomputed AlphaFold [48,49] structures were obtained for LAP59 (UniProt: Q57X92), LAP73 (UniProt: Q583W2), LAP102 (UniProt: Q581B5) and LAP173 (UniProt: Q585F7). For *T. brucei* LAPs 71 and 92 and *E. gracilis* LAP59, AlphaFold [48,49] predictions were computed using the DeepMind Colab Jupyter notebook [50] with default settings (Supplementary Figure S1).

Due to the size of LAP333 multiple approaches were required to model the structure. Firstly, GlobPlot [51] was used to identify globular domains within LAP333. Three globular domains were identified (Supplementary Figure S2) and their tertiary structures predicted using the AlphaFold DeepMind Colab Jupyter notebook [50] monomer model without the relaxation stage. The second LAP333 globular domain was divided into two sections due to computational restrictions (fragments 2A and 2B, Figures 4(b) and S3). These models are referred to as DeepMind monomer models. The second approach was to fragment LAP333 into N-terminal (residues 1–1681), C-terminal (residues 1682–3030) and an overlapping middle fragment (residues 1126–2480) (Supplementary Figure S4). These fragments were predicted using the ColabFold [52] AlphaFold instance with default settings (no relaxation) and are referred to as ColabFold [52] monomer predictions (Supplementary Figure S5). Thirdly, we predicted the full-length LAP333 structure using the AlphaFold [48,49] multimer [53] model with both the DeepMind Colab Jupyter notebook [50] and the ColabFold [52] instance, referred to as the DeepMind multimer and the ColabFold [52] multimer models respectively (Supplementary Figures S6–S8). For the DeepMind multimer model, the ‘use_multimer_for_monomers’ setting was selected, without relaxation and twenty recycles used. For the ColabFold [52] multimer model the settings were changed so a single model was computed, without relaxation and using 20 recycles. Models were visualized in either PyMOL [54] or iCn3D [55].

Protein structures were searched against PDB25 with DALI [56]. The top five hits with a Z score >2 were investigated using the DALI [56] structure viewer. Folds and domains were predicted for the query structure if the hit contained a domain or fold over the aligned region in InterPro [57]. To improve DALI searching for LAP333, AlphaFold models were fragmented into domain regions based on model structure. The same region across models was aligned in PyMOL [54] and where the RMSD was <5 only a single domain region was searched using DALI as above, excluding the LAP333 *trans*-membrane bundle (residues 2918–3030) which produced RMSD values >5 between models but only the DeepMind monomer model was searched. For LAP92, the metal-binding region was also extracted and searched (residues 703–784) with DALI [56] separately. All software used default settings unless otherwise stated.

### Comparative genomics and phylogenetics

Predicted proteomes were obtained for 36 organisms across the eukaryotic tree (see Supplementary Table S1 for details). The predicted proteomes were compiled as a single database and searched with *Trypanosoma brucei* 927 sequences using BLASTp [58] with an Expect value (E-value) threshold of 0.1. The top five hits per organism were filtered based on a calculated alignment length ≥30% coverage of the *T. brucei* query sequence length. Hits were used in reverse BLASTp searches. Orthology was predicted based on the top five hits with a calculated alignment length ≥30% of the query sequence identifying the original *T. brucei* query sequence. Alignment lengths were calculated based upon addition of the lengths of non-overlapping local alignments with gaps removed. Sequences from distantly related kinetoplastids *Blechomonas ayalai, Crithidia fasciculata, Leptomonas pyrrhocoris* and *Bodo saltans* were also used to search the database.

Predicted orthologs were aligned using MUSCLE [59] version 3.8.1551, trimmed using alncut [60] (version 1.06) with gaps only allowed in 25% of sequences per residue and approximate maximum likelihood phylogenetic trees constructed using FastTree [61] (version 2.1.10). Following confirmation of orthology, the unedited alignment was used to build an HMM profile to search through remaining organisms where no orthologs were identified (HMMER [62] version 3.2.1). The top five hits per organism were taken and used in reverse BLASTp against the original database with an E-value cutoff of 0.1. Orthology was considered if the top reverse BLASTp hit per query per organism identified one of the sequences in the HMM profile. Sequences were aligned, trimmed and a phylogenetic tree was built as described above to confirm orthology. The process was repeated until no additional sequences were identified.

Where orthologs were unidentified in Kinetoplastid organisms or an identified ortholog was incomplete, alternative strains were searched using either manual searching, batch_brb [63] or the TriTrypDB [64] orthology data with various kinetoplastid sequences as queries. Where this was also unsuccessful or the alternative strain had no predicted proteome data, additional tBLASTn [58,65] searches were performed against the genomes of these organisms using either TriTrypDB [64], Ensembl Protists [66] or the NCBI whole-genome shotgun contigs repository [67] with various kinetoplastid sequences as queries. Regions surrounding the identified tBLASTn hits were extracted, and Expasy [68] Translate was used to identify the ORF. If the protein sequence was identified across multiple reading frames due to unsequenced regions, these frames were fused to create a full-length protein. If the protein was identified across multiple reading frames, likely due to sequencing errors or a chimera created by assembly errors, original sequencing reads were downloaded and assessed – see Next-generation sequencing analysis. If this was the first identification of the protein, a reverse BLAST [58,65] was performed.

For LAP59, as many orthologs are predicted to contain the same domain (InterPro: IPR019176), additional searching of the InterPro database [57] was performed to identify all proteins containing the domain of interest. Results were filtered by domain topology and taxonomy restricted to groups where no ortholog was identified. The AlphaFold [48,49] predicted structures of these proteins were downloaded, analyzed in PyMOL [54], domain of interest extracted and aligned to the *T. brucei* AlphaFold structure domain using PyMOL [54]. Alignments which produced an RMSD of <3 were considered positive hits. The entire protein sequence was used in a BLASTp against the original organism database with an E-value cutoff of 0.1. Orthology was predicted if the top hit identified a previously identified ortholog. For LAP173, putative orthologs with a sequence length of <1000 residues were excluded to remove hits likely identified solely from the presence of a Sac3/GANP domain.

Additional best reciprocal BLAST (BRB) searches were performed against the EukProt TCS database [69] (excluding *Nonionella stella*) and assembled *B. saltans* and *P. confusum* transcriptomes using batch_brb [63] v1.0.1 with the top five hits and an alignment coverage of 30%. The EukProt TCS database (excluding *N. stella*) was searched with TbLAP333, *B. saltans* LAP333 fragmented gene predictions and hits from *Telonema sp. P-2* and *Colponema vietnamica* as queries. The *B. saltans* assembled transcriptome was searched with TbLAP333 as the query. The *P. confusum* transcriptome was searched with *P. confusum* LAP102 fusion protein (PCON_0077700). Identified transcripts of interest were translated using ExPasy [68] translate and included with the identified orthologs for phylogenetic analyses and for LAP333, additional HMMER searches.

Following identification of putative orthologs, sequences were aligned and edited as above (unless specified otherwise) and final maximum likelihood and Bayesian inference phylogenetic trees constructed using PhyML 3.0 [70] and MrBayes 3.2.6 [71] respectively. PhyML [70] was performed with default settings and a bootstrap of 1000. MrBayes was run on the CIPRES Science Gateway [72] portal with an MCMC generation of 800,000, 1000 sampling frequency with the first quarter as burn-in and a Γ shape rate variation with four categories.

### Next-generation sequencing analysis

Genomic or RNA-seq illumina reads were downloaded from the European Nucleotide Archive (ENA) (*A. deanei* genome: PRJEB36170 [73,74], *B. saltans* RNA-seq: PRJEB3146 [75,76], *E. monterogeii* RNA-seq: PRJNA680236 [77,78], *P. confusum* poly(A)-enriched RNA-seq: PRJNA414522 (sample SAMN07793202) [79,80]). Reads were assessed for quality, trimmed, base calls corrected and adapter sequences removed using fastp [81] version 0.23.2 with the following flags: —detect_adapter_for_pe, —overrepresentation_analysis, —correction and —cut_right. Quality assessment of trimmed reads was performed using FastQC (https://www.bioinformatics.babraham.ac.uk/projects/fastqc/) and MultiQC [82] version 1.13. Genome sequence files and GFF files were downloaded from TriTrypDB [64] versions 60 (*A. deanei* and *B. saltans*) or 61 (*E. monterogeii* and *P. confusum*). The genomes were indexed with BWA version 0.7.17 and reads mapped using BWA-MEM [83]. Reads were sorted by name with the -n flag, mates fixed (-m), resorted (without -n) and indexed using SAMtools [84] version 1.16.1. Mapped reads were visualized with JBrowse2 [85] version 2.2.1. Manual inspection was performed to identify indels in the genome sequences relative to the illumina reads. For *A. deanei* and *E. monterogeii* custom Python scripts (https://github.com/erin-r-butterfield/LAPs) were used to extract the genome sequence of the region of interest and correct identified indels. Expasy [68] Translate was used to translate the new sequence and identify the gene ORF. For *B. saltans* and *P. confusum*; the trimmed read names were altered with Awk (e.g. zcat ./trimmed/ERR152949_1.fastq.gz | awk ‘{{print (NR%4 = = 1) ? “@ERR152949_” ++i “/1”: $0}}’ | gzip -c > ERR152949_1.renamed.fastq.gz) [86] and the transcriptome assembled using Trinity v2.8.5 [87].

### Culture and *in-situ* tagging of *T. brucei*

Procyclic (PCF) *T. brucei* Lister 427 cells were cultured in SDM-79 medium supplemented with 10% (v/v) fetal bovine serum as described previously [28,88]. LAPs 71, 102 and 173 were C-terminally tagged *in situ* with either GFP or 3xHA using the pMOTag vectors [89]. LAP73 was N-terminally tagged *in situ* with a 12xHA tag using the p2929 vector [90]. We used the previously published NUP-1 [8], NUP-2 [9] and LAP59 [29] GFP tagged cell lines. Cell lines were maintained in required antibiotics at the following concentrations: 1 μg/mL puromycin, 25 μg/mL hygromycin and 10 μg/mL blasticidin.

### Immunofluorescence microscopy

Immunofluorescence microscopy was performed as described [91]. The following antibodies and concentrations were used: polyclonal rabbit anti-GFP (Santa Cruz) 1:1000, polyclonal rabbit anti-GFP (in house) 1:15,000, mouse anti-GFP (Roche) 1:3000, polyclonal rabbit NUP-1 anti-repeat (Covalab; NUP-1 peptide: NH_2_-CLNAAGVRVRTSQSDKD-COOH) 1:750 [8], monoclonal mouse MAb414 (anti-nuclear pore complex proteins) (Covance) 1:5000 [92,93], monoclonal rat anti-HA (Roche) 1:1000, monoclonal mouse anti-HA 1:1000 (Santa Cruz), polyclonal goat anti-rabbit Alexa Fluor 568 1:1000 (Life Technologies), polyclonal goat anti-mouse Oregon Green 488 1:1000 (Invitrogen), polyclonal goat anti-rat Alexa Fluor 568 1:1000 (Life Technologies). Widefield images were acquired with a Zeiss Axiovert 200 M inverted microscope with ApoTome.2 enabled and an Axiocam MRm camera. Apotome images were converted to conventional fluorescence images (without correction) using Zen Blue Lite Software (Zeiss). Confocal images were acquired with a Zeiss LSM700 inverted confocal microscope. Z-stack confocal images were 3D projected using the orthogonal projection tool within Zen Blue Lite. All images were set to minimum/maximum level within Zen Blue Lite and background and brightness adjusted with Photoshop (Adobe Inc.). Colocalization analysis was performed on widefield Apotome images converted to conventional fluorescence images (without correction) and set to minimum/maximum level (Zen Blue Lite Software). Pearson’s correlation coefficients were calculated for regions of interest using the BIOP JACoP plugin [94] in FIJI [95] with Otsu thresholding [96] and the fluorogram auto-adjusted on at least three cells for each stage of the cell cycle.

### Isolation and identification of lamina-interacting proteins

Protein–protein interactions were identified through co-immunoprecipitation as described [29,30]. Briefly, procyclic *T. brucei in-situ* GFP-tagged parasites were grown to a density of 2.5 × 10^7^ cells/mL. Parasites were harvested, flash frozen in liquid nitrogen and cryomilled using a Retsch PM100 planetary ball mill. Aliquots of the resulting frozen grindate were resuspended in various extraction buffers (LAPs 59 and 71: 20 mM HEPES, pH7.4, 250 mM NaCl and 0.5% Triton; LAP102: 20 mM HEPES, pH7.4, 250 mM NaCl and 0.5% CHAPS; NUP-1: 20 mM HEPES, pH 7.4, 250 mM NaCl, 0.5% Triton and 0.5% deoxy-BigCHAP; NUP-2: 20 mM HEPES, pH 7.4, 250 mM Citrate, 0.5% Triton) containing a protease inhibitor cocktail without EDTA (Roche). These were sonicated on ice with a microtip sonicator (Misonix Ultrasonic Processor XL) at Setting 4 (~20 W output) for 2 × 1 second to break apart aggregates that may be invisible to the eye, and clarified by centrifugation (20,000 x *g*) for 10 min at 4°C. Clarified lysates were incubated with magnetic beads conjugated with polyclonal anti-GFP llama antibodies on a rotator for 1 h at 4°C. The magnetic beads were harvested by magnetization (Dynal) and washed three times with extraction buffer prior to elution with 2% SDS/40 mM Tris pH 8.0. The eluate was reduced in 50 mM DTT and alkylated with 100 mM iodoacetamide, prior to downstream mass spectrometry (MS) analyses using either electrospray ionization (ESI) (NUP-1, NUP-2 and LAP102) or Matrix-Assisted Laser Desorption – Time of Flight (MALDI-TOF) (LAP59 and LAP71). Eluates from the affinity capture experiments were loaded into the wells of a 5% acrylamide gel and run at 100 V for 5 minutes to allow the proteins to migrate approximately 2 mm into the gel (for ESI) or fractionated using SDS-PAGE (Novex 4–12% Bis Tris gels (Life Technology)) (for MALDI-TOF). The gels were then fixed for 5 minutes in 50% methanol/7% acetic acid, and then stained using GelCode^TM^ Blue Stain (Thermo Scientific). The protein bands were excised from acrylamide gels and destained using 50% acetonitrile, 40% water, and 10% ammonium bicarbonate (v/v/w). Gel pieces were dried and resuspended in trypsin digestion buffer; 50 mM ammonium bicarbonate, pH 7.5, 10% acetonitrile, and 0.1–2 μg sequence-grade trypsin, depending on protein band intensity. Digestion was carried out at 37°C for 6 hours prior to peptide extraction using C18 beads (POROS) in 2% TFA (trifluoroacetic acid) and 5% formamide. Extracted peptides were washed in 0.1% acetic acid (ESI) or 0.1% TFA (MALDI-TOF) and analyzed on a LTQ Velos (ESI) (Thermo) or pROTOF (MALDI-TOF) (PerkinElmer). The MALDI-TOF data was analyzed using ProFound [97], and the ESI LC-MS data analyzed using the Global Proteome Machine [98]. Identified proteins were ranked by peptide log intensity and the top 50 hits selected for further analyses.

### Modeling of protein complexes

LAP333 DeepMind monomer fragments were modeled with LAP59 using AlphaFold DeepMind Colab Jupyter notebook [50] multimer modeling [53] with 20 recycles and no relaxation. Models were visualized in PyMOL [54]. Electrostatic charges were determined using the APBS plugin [99,100] with default settings and hydropathy visualized using the color_h PyMOL script [101,102].

## Results

### Identification of candidate trypanosome lamina-associated proteins

**Figure 1.**
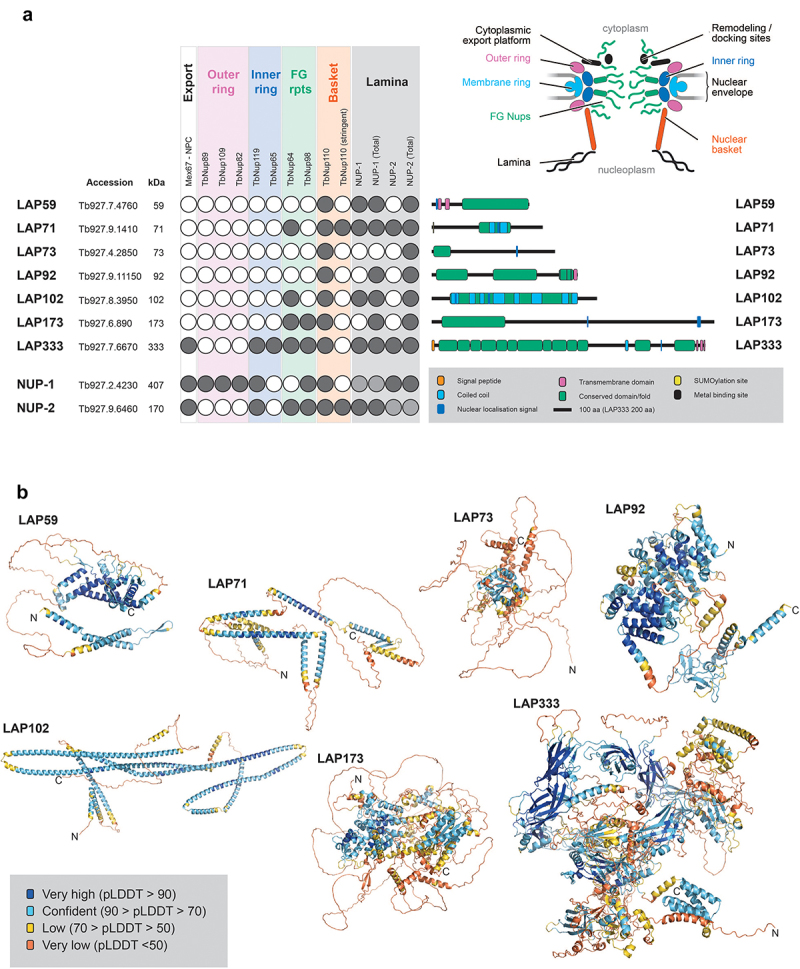
Identification of lamina-associated proteins (LAPs). (a) NUP-1 and NUP-2 were C-terminally tagged and used as handles in co-immunoprecipitation. The data were cross referenced against previously published NUP-1, NUP-2 and NPC co-immunoprecipitations [9,29], identifying seven proteins interacting with both the lamina and the NPC. Dark gray and white circles indicate presence or absence in co-immunoprecipitations respectively, light gray indicates a self-identification. Total refers to the analysis of the entire immunoprecipitation rather than selected bands. Stringent refers to high-stringency conditions. Colored boxes for the circle plot indicate the region of the NPC and match to the inset NPC figure. Colors on the LAP schematics are shown in the figure legend. *In silico* analysis of LAPs structures identified several domains, shown as green boxes, including a cytochrome B561 domain in LAP59, provisional chromosomal segregation domain in LAP71, a Nup35/53-type RNA-binding domain in LAP73, mitochondrial associated sphingomyelinase and metal-binding domain in LAP92, an SMC domain in LAP102, a Sac3/GANP domain in LAP173 and up to 13 Ig-like folds in LAP333. (b) AlphaFold [48,49] predicted structures for the LAPs are colored by pLDDT for confidence as indicated. For LAP333 fragmented and full-length structures were predicted individually using the monomer [48] and multimer [53] models respectively with the DeepMind [50] and ColabFold [52] notebooks. The DeepMind multimer model is shown. Additional LAP333 fragment and full-length models are in Figure 4 and Supplementary Figures S5 and S6.

There is considerable divergence between the trypanosome lamina and that in other lineages, which extends beyond core components, as evidenced by proteomics and high-throughput localization studies not limited to the distinct lamina system [3,9,26,28,29,103–105]. To increase understanding of the NE/NPC/lamina nexus, we exploited a targeted strategy based on physical association with known NE components. We performed co-immunoprecipitation on cryomilled cell lysates from NUP-1:GFP and NUP-2:GFP cell lines and analyzed with LC-MS (ESI) to identify additional lamina protein–protein interactions (PPIs) (Supplementary Tables 2 and 3). These were sorted by peptide log intensity, and we selected the top 50 hits, cross-referencing to data from multiple NPC and lamina immunoisolations [9,29] to robustly identify new proteins from both a lamina and an NPC purification. This strategy identified seven proteins as both lamin and NPC PPIs. We designate these proteins as lamina-associated proteins or LAPs (Figure 1).

**Figure 4.**
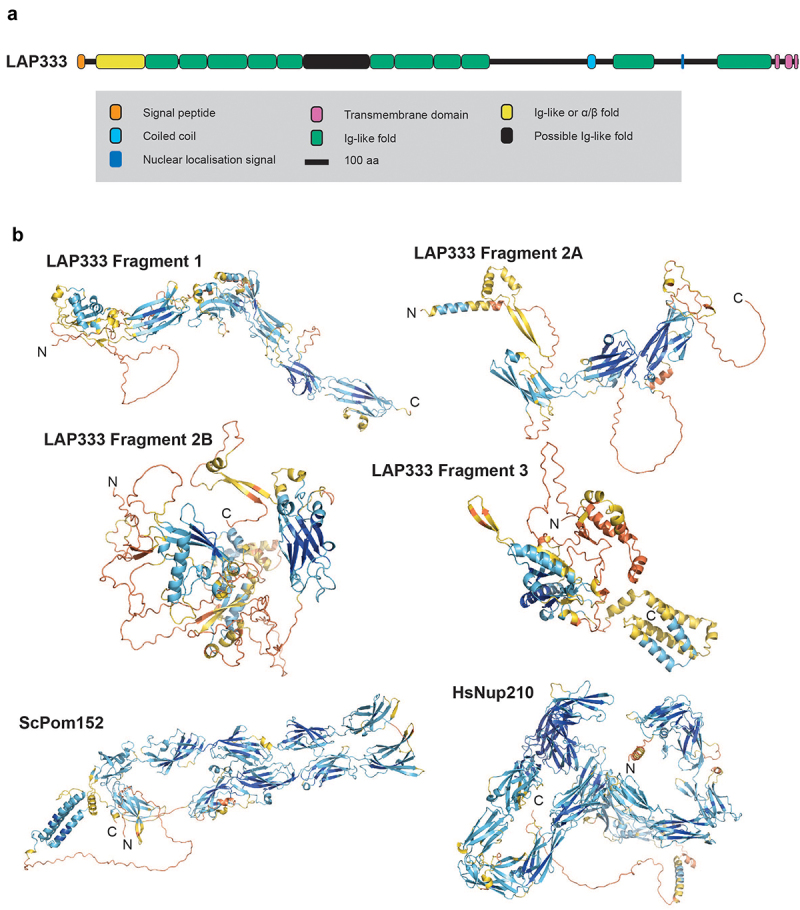
Comparison of LAP333 structure with *S. cerevisiae* Pom152 and *H. sapiens* Nup210. (a) Schematic of LAP333 highlighting the Ig-like folds (colored as per legend). (b) The DeepMind monomer models for LAP333 and AlphaFold models for the membrane ring protein analogs ScPom152 and HsNup210 colored by pLDDT as per Figure 1. The ScPom152 and HsNup210 precalculated structures were downloaded from the AlphaFold database [49].

**Figure 2.**
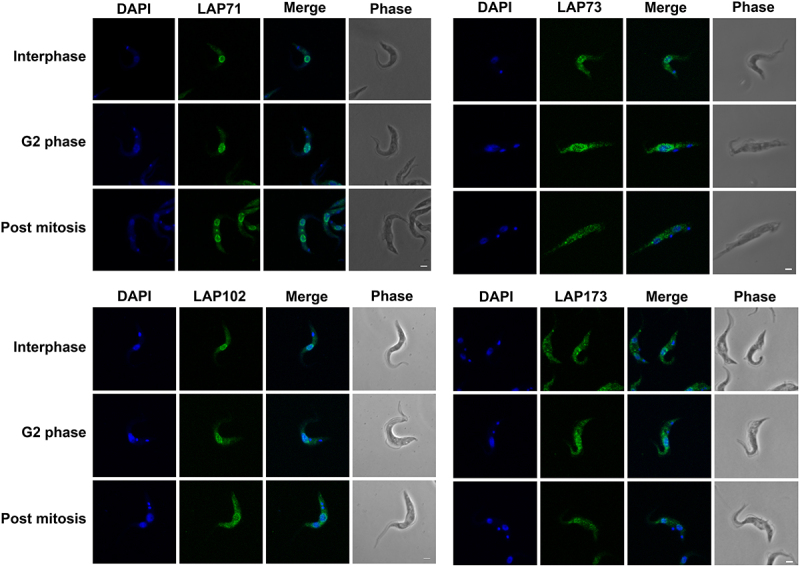
LAPs are localized to the NE throughout the cell cycle. LAPs were visualized by *in situ* tagging and immunofluorescence microscopy. Images shown are the 3D projection of confocal z-stacks. LAP71 and 173 were C-terminally tagged with GFP, LAP73 was N-terminally tagged with 12x HA and LAP102 was C-terminally tagged with 3x HA. Scale bar = 2 µM. The LAPs show NE staining throughout the cell cycle with an additional inter nuclei bridge for LAP102 post mitosis.

To validate this cohort as *bona fide* lamina-associated and/or NE proteins, each LAP gene was tagged *in situ* and the resulting protein chimera localized using immunofluorescence microscopy (Figure 2). We were unable to tag LAP92 [29] or LAP333, and indeed high-throughput and other studies similarly failed to deliver clear localizations for either protein [29,104–106]. We previously localized LAP59 to puncta on the nuclear rim and the Golgi complex [29]. LAP59 possess an N-terminal *trans*-membrane domain and nuclear localization signal (NLS), suggesting that LAP59 is embedded in the NE. LAP71 and 73 localize to puncta at the nuclear periphery throughout the cell cycle and between daughter nuclei during mitosis, similar to NUP-1 [8]. LAP102 localizes to the NE, but during anaphase forms a punctate bridge between the two daughter nuclei (Figure 2) while LAP173 localizes primarily to puncta at the NE but is also present in the nucleoplasm. The localizations for LAP59, 71, 73, 102 and 173 are consistent with high-throughput data [104,105]. Hence, we were able to validate five of the cohort as present at the NE, *albeit* in some cases detecting additional locations within the nucleoplasm, ER or Golgi complex, which is similar to many mammalian NE proteins [107].

### LAPs represent a diverse cohort of proteins

We used multiple *in silico* algorithms to analyze LAP sequences for informative structural/sequence features (Figure 1(a)). With the exception of LAP92, all the LAPs are predicted to contain a likely monopartite NLS, while LAP102 contains a second monopartite NLS and LAP173 contains an additional bipartite NLS. LAP59 is predicted to contain two N-terminal *trans-*membrane domains and a C-terminal cytochrome B561 domain (Supplementary Table S4). LAP59 has been observed to be essential in some stages of the *T. brucei* life cycle [108].

LAP71 contains an N-terminal SUMO-interacting motif (residues 5–11) [109], is SUMOylated at K_228_ [110] and is predicted to contain two coiled-coil regions. A provisional chromosomal segregation domain and similarity to several proteins involved with microtubules, spindle formation, cell cycle and other functions was also identified but restricted to the coiled-coil regions. A possible cell cycle function for LAP71 is supported by identification of LAP71 as a PPI of KKT-interacting protein 1 (KKIP1) although localization suggests it is not part of the kinetochore [111]. LAP71 is not cell cycle regulated [112], and although essentiality has been noted [108], knockdown does not induce major cell cycle defects [113] (Supplementary Figure S9).

LAP73 contains an Nup35/Nup53-type RNA-binding domain. In yeast, Nup53 (ScNup53/59) is involved with anchoring the pore to the NE through an amphipathic lipid packing sensor (ALPS) motif. *T. brucei* contains a Nup53 ortholog (TbNup65) but uses a *trans*-membrane domain instead [29]. While a Nup35/Nup53-type domain could suggest orthology with ScNup53/59, the absence of interactions with the NPC inner ring [29] or an obvious membrane anchor suggests otherwise. LAP73 is essential for some stages of the *T. brucei* lifecycle [108].

**Figure 3.**
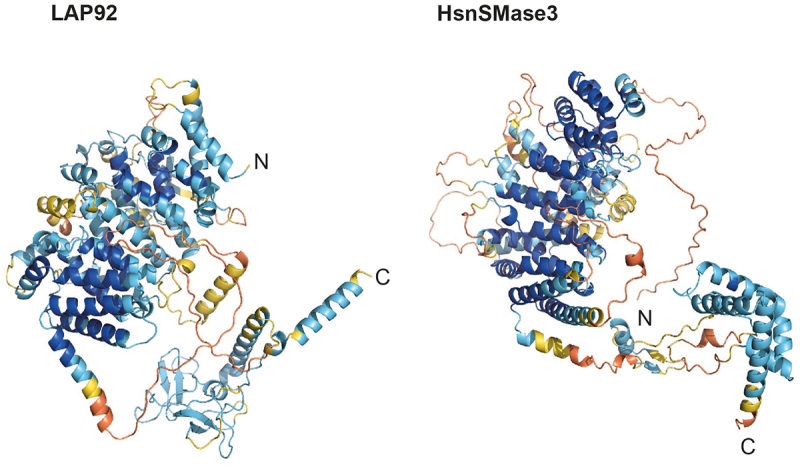
AlphaFold models for LAP92 and HsnSMase3. The domain topology for LAP92 suggests similarity with *H. sapiens* nSMase3. The structures for the two proteins are shown and colored by their pLDDT as per Figure 1. The precalculated structure for HsnSMase3 was downloaded from the AlphaFold database [49]

LAP92 possess an N-terminal mitochondrial sphingomyelin phosphodiesterase domain, an armadillo-type fold, a likely Zn^2+^-binding domain (with conservation of the metal-binding sites: Cys708, Cys711, Cys745, Cys748, Cys769 and Cys772) and a C-terminal *trans*-membrane domain. Domain predictions suggest LAP92 is a structural homolog of the *H. sapiens* neutral sphingomyelinase 3 (nSMase3), a Mg^2+^ or Mn^2+^-dependent enzyme involved in catabolism of sphingomyelin to ceramide [114]. HsnSMase3 localizes to the endoplasmic reticulum, Golgi, Golgi-associated apparatus and outer nuclear envelope [107,114–117], interacting with several nucleoporins including HsNup35 [116,117]. Knockdown results in altered mitotic NE dynamics and post-mitotic insertion of NPCs [117,118]. LAP92 interacts with Nup110 rather than Nup65 (the HsNup35 ortholog) [29,116] and is essential [108]. Although LAP92 and HsnSMase differ in their specific interactions and the metal-binding domain [115], conservation of protein size, domain architecture, structure (Figure 3), localization pattern [119] and NPC interaction [9,29,116] suggests structural and functional homology between LAP92 and nSMase3.

LAP102 has extensive coiled-coil regions and an overlapping SMC-domain but is clearly not a canonical SMC component as it lacks additional features [120] (Figure 1(b)). LAP102 expression peaks during S-phase [113,121] and knockdown generates cells with reduced DNA content (< 2C, where C is haploid DNA) [113] (Supplementary Figure S9), although it is nonessential [108].

LAP173 is predicted to contain a Sac3/GANP domain which may suggest that this protein forms part of the TREX-2 complex (Sac3-Thp1-Sem1-Sus1-Cdc3), but as no additional TREX-2 components have been identified, this is the sole representative of this mRNA maturation complex [122]. The identification of LAP173 as Sac3 is supported by interactions with FG-Nups 64 and 98, as these proteins contain a similar repeat type to the *S. cerevisiae* FG-Nups 1 and 60 which interact with ScSac3 [29,123–125]. Moreover, ScNup1 is required for the localization of ScSac3 to the NPC [124]. Knockdown of LAP173 suggests it is essential during multiple stages of the trypanosome life cycle [108].

LAP333 contains an N-terminal signal peptide, multiple C-terminal *trans-*membrane domains, a coiled-coil region and up to 13 immunoglobulin-like (Ig-like) folds (Figures 1 and 4), suggesting anchoring in the NE. LAP333's architecture and protein interactions suggest LAP333 as a structural homolog to the NPC membrane ring proteins Nup210 and Pom152 from humans and yeast, respectively. *Xenopus laevis* GP210 (the ortholog of HsNup210) contains an N-terminal *trans*-membrane domain, 15 Ig-like folds, a β-strand rich C-terminal domain and a C-terminal *trans*-membrane domain [126] while ScPom152 contains three N-terminal *trans*-membrane domains followed by ten Ig-like folds [127–129]. ScPom152 interacts with Nup157 and Nup170 [130]. TbNup119 (an ortholog of ScNup157 and ScNup170) interacts with LAP333 [29,131], supporting the designation of LAP333 as a structural and possible functional homolog of ScPom152. Significantly, LAP333 also interacts with TbNup65, a protein likely involved in NPC anchoring due to its orthology with ScNup53 and possession of a *trans*-membrane domain [29,132], further supporting the involvement of LAP333 in NPC anchoring. Identification of LAP333 as a NUP-2 interactor and the highly similar interactomes of LAP333 and NUP-2 [9,29] suggests these proteins closely interact and act as an additional anchoring point between the lamina and the NPC [9]. LAP333 together with LAP59 interacts with the kinetochore protein KKT18 [106] and consequently may also indicate that KKT18 interacts closely with the nuclear envelope during G1.

### The majority of LAPs are kinetoplastid specific

We performed phylogenetic analysis to understand LAP origins and evolution (Figure 5, Supplementary Tables S4–S6, Supplementary Figures S10–S19). LAP orthologs were identified through best reciprocal BLAST (BRB) and iterative HMMER. Many LAP sequences were incomplete necessitating additional analyses, transcriptome reassembly and searching (Supplementary results).

**Figure 5.**
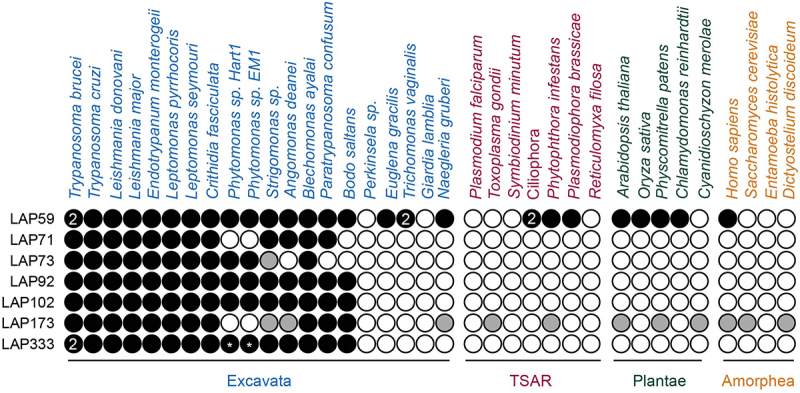
Distribution of LAPs across the eukaryotes. Black circles indicate an ortholog identified, gray circles indicate low confidence hits. White circles indicate no hit identified, numbers indicate the number of orthologs identified and *indicates incomplete sequences. *Strigomonas sp*. indicates *S. culicis* or *S. galatii* and Ciliophora indicates *T. thermophila* or *S. coeruleus –* full details in Supplementary Table S5. Additional LAP333 hits were identified in the TSAR organisms *Telonemia subtile, Telonemia sp. P-2, Colponemia vietnamica, Colpnemia sp. Colp-10* and *Colponemia sp. Colp-15*, however, as these organisms were cocultured in the presence of kinetoplastids these hits likely represent contamination and are therefore not shown. Orthologous sequences and sources are provided in Supplementary Tables S1, S5 and S6.

The majority of LAPs (71, 73, 92, 102 and 333) are restricted to the Kinetoplastids. No LAP71 orthologs were identified in *Phytomonas* or *B. saltans*. Absence from *Phytomonas* may be due to incomplete sequencing, lineage-specific losses or high divergence of the protein, while the *B. saltans* absence could suggest LAP71 acquisition occurred in association with parasitism, although we cannot eliminate the possibility of incomplete genome data. LAP71 orthologs were identified in *A. deanei* and *S. culicis* but we were unable to resolve their position phylogenetically, due to divergence (Supplementary Figure S10).

LAP73 is restricted to the Trypanosomatida (Supplementary Figure S11) although no ortholog was identified in *Paratrypanosoma confusum*. A potential homolog is present in *S. culicis* although it is unclear if this is a true ortholog of LAP73 as the N-terminal RRM domain has a low probability of being a Nup53-type (HHPred: 17^th^ hit, 41% probability), there is limited conservation of the sequence with the remainder of the Kinetoplastids (Supplementary Figure S12) and no corresponding ortholog was identified in *A. deanei*. The absence of a LAP73 ortholog in *A. deanei* could suggest either incomplete sequencing or a lineage-specific loss of this protein. The absence of an ortholog in *P. confusum* and *B. saltans* may suggest a late origin of LAP73, although once more we cannot exclude the possibility of incomplete sequence data. No orthology was detected between LAP73, TbNup65, ScNup53/59 and HsNup35. Combined with the absence of identified interactions between LAP73 and the NPC inner ring, it is unclear if LAP73 is directly involved with the NPC structural core. LAP92 is present across the Kinetoplastids (excluding *Perkinsela*) (Supplementary Figure S13). LAP92 shows no homology to the functional nSMase identified in *T*. brucei [133], consistent with HsnSMase3, which shows no homology to other *H. sapiens* nSMases [114].

LAP102 is present across the Kinetoplastids (excluding *Perkinsela*), with high levels of conservation, necessitating a decrease in editing frequency (gaps allowed in 75% of sequences) to ensure sufficient signal for phylogenetic reconstruction (Supplementary Figure S14). *P. confusum* has several insertions relative to other Kinetoplastid sequences (Supplementary Figure S15).

The LAP333 structure prediction indicates similarity between LAP333, ScPom152 and HsNup210; we therefore performed additional BRB searches against the EukProt TCS database [69] (excluding *Nonionella stella*) to confirm kinetoplastid restriction (Figure 5, Supplementary Figure S16). Although additional hits were detected in TSAR, this is likely due to contamination with kinetoplastid sequences [134,135] (Supplementary results), supporting the kinetoplastid specificity of LAP333. We could identify at least partial LAP333 sequences in the Trypanosomatida, Eubodonida and Parabodonida, suggesting LAP333 may have been acquired early in Kinetoplastid evolution and was since lost from the Neobodonids.

**Figure 6.**
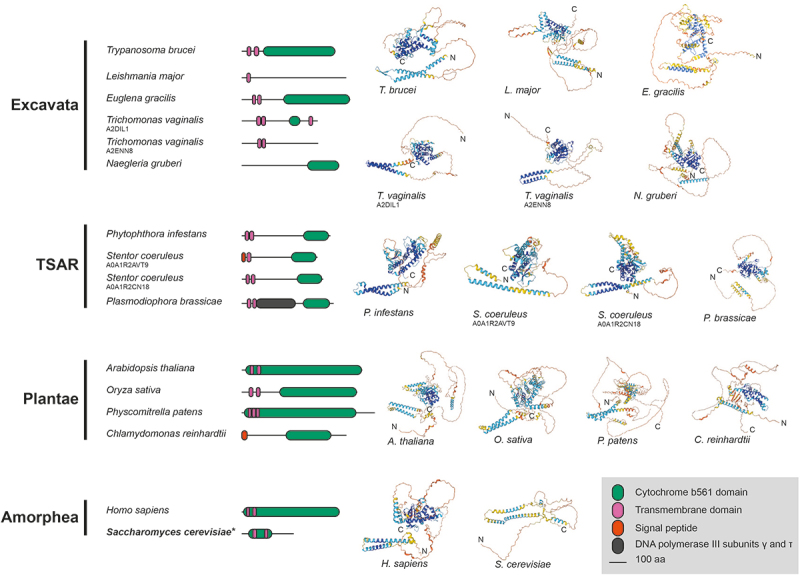
Schematics and structures of representative LAP59 orthologs compared to ScPom34. LAP59 was detected across the eukaryotes with the orthologs showing similar structures and domain topology. Colors on the schematics are shown in the Figure legend. *indicates *S. cerevisiae* Pom34 was not detected as an ortholog of LAP59 but has been suggested as a putative ortholog of *H. sapiens* TMEM209 [138], itself a LAP59 ortholog. Precalculated structures were downloaded from the AlphaFold database [49] excluding *E. gracilis* which was calculated with [50]. All structures are colored by pLDDT as per Figure 1.

Contrastingly, LAP59 and LAP173 are detected across the eukaryotes. LAP59 orthologs are architecturally conserved, with N-terminal *trans*-membrane domains and a C-terminal cytochrome B561 domain predicted in the majority of orthologs (Figure 6, Supplementary Table S4, Supplementary Figure S17). The absence of a predicted LAP59 ortholog in *Perkinsela* and *Giardia lamblia* may be due to their reduced gene content [136,137]. BRB and iterative HMMER failed to identify alveolate LAP59 orthologs but additional searches of the InterPro [57] database identified orthologs in the ciliate *Stentor coeruleus*. The *Homo sapiens* TMEM209 (Transmembrane protein 209) and *Arabidopsis thaliana* PNET1 proteins were also identified as orthologs of LAP59. TMEM209 is a putative ortholog to the *S. cerevisiae* NPC membrane ring protein ScPom34 [138] which interacts with the NPC in lung cancer cells [139], shows colocalization with the NPC and has been suggested as an additional NPC component [140], while PNET1 is a membrane ring nucleoporin [141]. Together this evidence suggests that LAP59 is also a membrane ring nucleoporin and is supported by the PPIs [29], LAP59 structural predictions and similar localizations between LAP59, TMEM209 [107,139,140,142] and PNET1 [141]. Although LAP59 and ScPom34 share similar domain topology, we did not identify ScPom34 as an ortholog of LAP59. This is supported by the AlphaFold [48,49] models, which suggest the two proteins have distinct structures (Figure 6).

The presence of a Sac3 domain in LAP173, necessitated additional filtering of the LAP173 hits (sequences > 1000 aa) to prevent misidentification of Sac3 domain-containing proteins as LAP173 orthologs. No *Phytomonas* orthologs were identified which may suggest a lineage-specific loss. Phylogenetic analysis of the Kinetoplastid sequences identified the *S. culicis* and *A. deanei* sequences as an outgroup to the remainder of the kinetoplastids, making orthology predictions unclear for these two organisms. We did identify several LAP173 homologs outside of Kinetoplastida but due to sequence divergence we were unable to resolve them phylogenetically. While the major regions of conservation are within the Sac3 domain, additional conservation is present at the C-terminus (Supplementary Figures S18 and S19), supporting assignment as possible LAP173 orthologs. Finally, identification of *A. thaliana* and *S. cerevisiae* Sac3 [124,143] as possible LAP173 orthologs supports the designation of LAP173 as Sac3.

### LAPs interact with both the NPC and lamina

**Figure 7.**
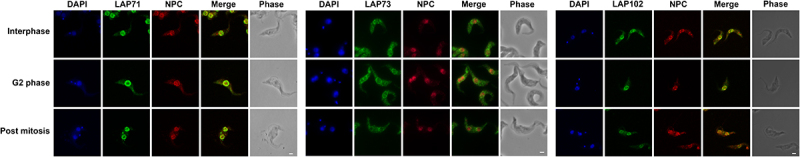
LAPs show limited colocalisation with the NPC. Epitope tagged LAPs were visualized with immunofluorescence microscopy against the NPC. LAP71 and 102 were C-terminally tagged with GFP and 3x HA respectively. LAP73 was N-terminally tagged with 12x HA. The NPC was visualized using MAb414 against the FG repeats (red). Images show 3D projection of confocal z-stacks for LAP71 and 102 and Apotome widefield images of LAP73 in green. Scale bar = 2 µM. Although LAPs exhibit NE staining, there is limited colocalization of LAPs 71 and 102 with the FG Nups, while LAP73 shows no colocalization with the FG Nups (Supplementary Figure S20).

**Figure 8.**
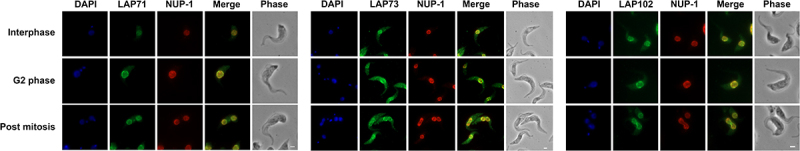
LAPs show some colocalisation with NUP-1. Epitope tagged LAPs were visualized with immunofluorescence microscopy against NUP-1. LAP71 and 102 were C-terminally tagged with GFP, LAP73 was N-terminally tagged with 12xHA. NUP-1 was visualized using an antibody against the repeat region of the protein (red). Images show Apotome widefield images of LAP71, 73 and 102 respectively (green). Scale bar = 2 µM. Some overlap is visible between the LAPs and the NUP-1 repeat, although no overlap is seen between the LAP102 and the NUP-1 internuclear mitotic bridge (Supplementary Figure S21).

We investigated relationships between the LAPs, the lamina and the NPC in more detail. We selected three LAPs, specifically 71, 73 and 102 as we were unable to assign functions from structural and phylogenetic data and compared their locations with the lamina and NPC. We visualized the NPC using the MAb414 antibody which binds to the NPC FG repeats [92,93]. Some colocalization was observed between the NPC and LAPs 71 and 102 (Figure 7, Supplementary Figure S20) supporting the interaction data. No colocalization was observed for LAP73 suggesting this is not proximal to the NPC (Figure 7, Supplementary Figure S20). We also compared LAPs 71, 73 and 102 to NUP-1 using an antibody raised against the NUP-1 central repeats. Widefield images indicated some colocalization between LAPs 71, 73 and 102 with NUP-1 (Figure 8, Supplementary Figure S21). Additionally, although NUP-1 is present as an umbilicus between separating nuclei during anaphase it does not colocalize with LAP102 (Figure 8, Supplementary Figure S21).

**Figure 9.**
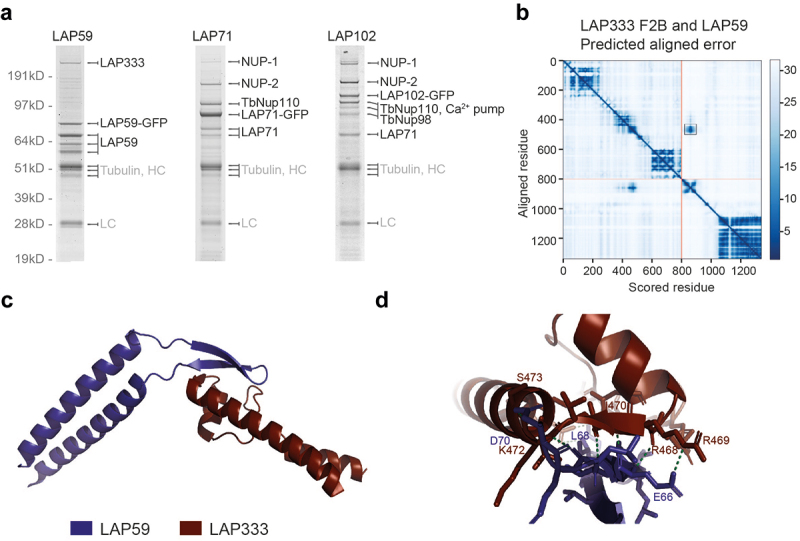
LAP interactors. (a) LAP59, 71 and 102 were tagged with GFP and used as handles in co-immunoprecipitations using either cut bands with MALDI-TOF (LAP59 and 71) or total precipitates and ESI (LAP102). A full list of LAP102 interactors is in Supplementary Table S7. AlphaFold DeepMind multimer [48–50,53] modeling supports an interaction between the LAP333 F2B fragment and LAP59 as shown by the predicted aligned error plot (b). Red lines indicate the end of the LAP333 F2B sequence. The black box highlights the high confidence region and a model of the region is shown in (c). Proteins are colored by chain as per the legend. (d) Expansion of interacting region showing interactions (green dashed lines) within 3.5 Å. LAP333 F2B and LAP59 colored as in (C).

As immunofluorescence microscopy suggests interactions (*albeit* indirect in some cases) between LAPs, NUP-1 and the NPC, we performed co-immunoprecipitation using LAPs 59, 71 and 102 to identify PPIs. LAP59 was chosen to investigate the membrane ring nucleoporin assignment, while LAPs 71 and 102 were chosen to identify interacting partners to assign putative functions. Proteins were C-terminally tagged *in situ* with GFP and co-immunoprecipitated from cryomilled cell lysates and subjected to mass spectrometry [29,30]. LAP59 identified itself and LAP333 (Figure 9), further support for LAP333 as a membrane ring Nup and structural homolog of Pom152 and Nup210. The three forms of LAP59 identified likely represent post-translational modifications and/or proteolysis. DeepMind multimer [53] modeling predicts an interaction between LAP59 and LAP333, identifying a region between the LAP59 *trans*-membrane domains (residues 54–71) and a region partially overlapping the LAP333 coiled-coil (residues 2128–2165) as a possible interaction site (Figures 9(b,d) and S22–S25) through formation of parallel β-sheet interactions (residues: LAP333: 453, 468–470, 472, 473, 482 and LAP59: 61, 66, 68, 70). Electrostatic charge and hydrophobicity are compatible with the predicted interaction (Supplementary figure S26) although some stereochemical clashes are present, likely due to a lack of Amber relaxation in model generation [48].

**Figure 10.**
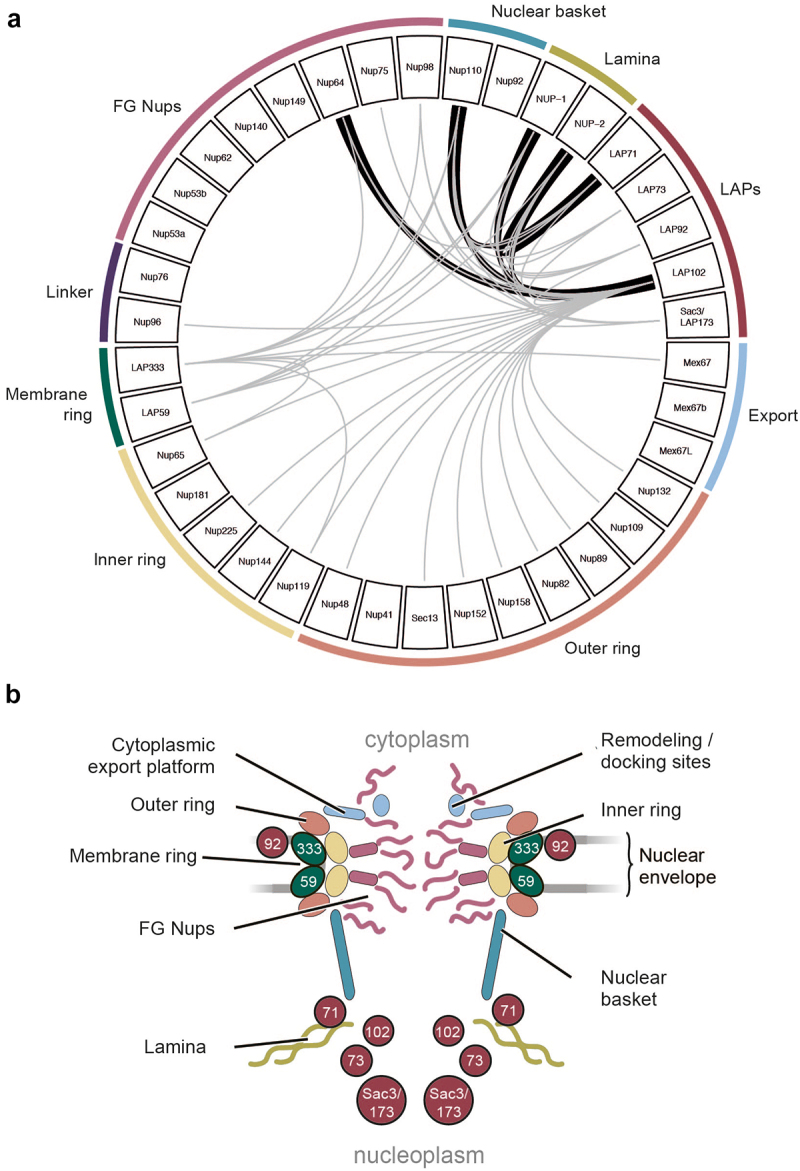
Model of the *T. brucei* NE. (a) Summary of new and published LAP interactions [9,29]. Grey lines indicate single direction identification, thick black lines indicate reciprocal identification showing the LAPs primarily interact with the NPC basket, inner and outer rings and the lamina. (b) Stylized model of the *T. brucei* NE colored as per (A) and predicted locations for LAPs.

Stringent extraction conditions to identify PPIs identified NUP-2 and Nup110 (a NPC basket protein) as LAP71 and LAP102 interactors (Figure 9). LAP102 also identified additional PPIs, including NPC subunits, additional LAPs and NUP-1 (Supplementary Table S7, Figure 10). As LAP71 and 102 are coiled-coil proteins in close proximity to the lamina and the NPC, these may interact with Nup92 and Nup110, supported by reciprocal identifications of Nup110 and NUP-2 for LAP71 and LAP102 [9,29], and identification of LAP71 as a PPI for Nup110 in stringent conditions [9] (Figures 1, 9 and 10). In yeast, the nuclear basket is composed of Mlp1, Mlp2, Nup60, Nup1 and Nup2 [131]. While LAPs 71 and 102 are coiled-coil proteins like Mlp1/2, they are considerably smaller (71 kDa and 102 kDa vs. 219 kDa and 195 kDa respectively) and Nups 110 and 92 have been proposed as the Mlp analogs in *T. brucei* [29,144], but it is possible that the *T. brucei* nuclear basket contains more coiled-coil subunits than *S. cerevisiae*.

Overall, the LAPs primarily interact with the lamina, NPC nuclear basket and the inner and outer NPC ring subunits [9,29] (Figures 1 and 10). Few FG-Nup PPIs were identified (4/9 FG-Nups) (here and [29]) and where identified are restricted to multi-complex FG-Nups attached to the outer ring or a component of the outer ring itself [29]. Additionally, only a single PPI was identified between a LAP (LAP333) and the export system(1/3) [29,145], which is consistent with a location at the membrane ring and not the transport channel.

## Discussion

We have identified seven trypanosome NE proteins, or LAPs, that interact with both the lamina and the NPC. For five of the cohort, we demonstrate a presence at the NE by microscopy, while proteomic analysis both here and previously [9,29] identified all as interactors in two or more immunoisolations using lamina and NPC proteins as affinity handles. We consider these identifications as robust and extend understanding of the composition of the trypanosome NE considerably.

The structures and interactomes of LAP59 and 333 suggests much greater structural conservation of the NPC membrane ring than previously considered [131]. The presence of LAP59 across eukaryotes suggests an ancient origin and presence in LECA. Furthermore, a similar localization for LAP59 orthologs in multiple organisms also supports a conserved function in anchoring the NPC. The *H. sapiens* LAP59 ortholog (TMEM209) may interact with HsNup205 [139], but current *H. sapiens* NPC models do not include TMEM209 [146]. Similarly, PNET1 (*A. thaliana* LAP59 ortholog) interacts with the NPC, primarily the inner and cytoplasmic rings [141]. Significantly, LAP333 has architectural similarities to *H. sapiens* and *S. cerevisiae* luminal ring proteins Nup210 [146] and Pom152 [130] respectively, specifically a signal peptide, multiple Ig-like folds and *trans*-membrane domains *albeit* in the absence of sequence similarity and differing domain topology. The remarkably similar architectures make the possibility of convergent evolution highly unlikely and is further supported by the different domain topologies between Nup210 orthologs [147,148]. Nup210 is broadly conserved, with orthologs present in plants [149], TSAR [147] and the Excavates [148] and hence likely present in LECA [148], but there are many lineages lacking an identifiable Nup210 ortholog including fungi [138], some algae [18], kinetoplastids [29] and apicomplexa [150–152] likely representing secondary losses [148] or as our current data suggests, loss of sequence similarity but retention of structural homology. Current models of the *S. cerevisiae* and *H. sapiens* NPC suggest arrangement of ScPom152 and HsNup210 within the NPC are somewhat distinct, with ScPom152 anti-parallel dimers forming arches between the spokes while HsNup210 forms butterfly structures composed of eight copies of HsNup210, *albeit* that the overall placement of subunits is conserved [126,130,146]. The domain arrangement of LAP333 may suggest a further variant pore anchor structure. Furthermore, *S. cerevisiae* Pom34 interacts with the Pom152 *trans*-membrane domain [130] and contrasts with the predicted LAP333 and LAP59 interaction site, between a LAP59 inter *trans*-membrane domain β-sheet and a region overlapping the LAP333 coiled-coil region.

LAP92 is structurally homologous to *H. sapiens* nSMase3 based on clear structural similarities. HsnSMase3 is involved in remodeling the NE following mitosis and postmitotic NPC insertion [118] and suggested via modulating local ceramide levels at the nuclear pore [118]. As *T. brucei* undergoes closed mitosis [131] insertion of new NPCs likely follows a pathway similar to interphase assembly [153] for which ceramide synthesis may be important [154]. Although similar to HsnSMase3, LAP92 has diverged within the metal-binding domain and PPIs and hence functional equivalency remains unclear.

LAP173 is a Sac3 ortholog, containing both a Sac3 domain and similar PPIs, including nuclear basket and FG-Nups [29]. Sac3 is a TREX-2 component which in yeast is composed of Sac3, Thp1, Cdc31, Sem1 and Sus1 and interacts with the nuclear basket [122]. Trypanosomes possess a divergent RNA export platform, utilizing three Mex67 paralogs [145] and no canonical cytoplasmic RNA export platform [29,122,131,155], although post-nuclear export regulation is present [156]. Sac3 in trypanosomes may represent a conserved core for anchoring mRNA-processing components in the NPC vicinity but with much of the associated apparatus lineage-specific and apparently dispensable [155].

LAP71, 73 and 102 are lineage-specific proteins but represent additional trypanosome NPC and lamina components. LAP71, a coiled-coil protein, interacts with the nuclear basket component Nup110 and lamina protein NUP-2, and may act to extend the basket and/or connect the NPC to the lamina. LAP102, a coiled-coil protein, interacts with both NUP-1 and NUP-2 also in the vicinity of the NPC basket protein Nup110. LAP73 is predicted to contain a Nup53-type RNA-binding domain, but the lack of conserved interactions and no obvious membrane anchor suggests LAP73 is not orthologous to yeast or animal Nups.

Restriction of the majority of the LAP cohort to kinetoplastids further highlights the diversity within the *T. brucei* NE. Absence of recognizable LAP orthologs in *E. gracilis* suggests these proteins were acquired following divergence from the Euglenoida; however, the presence of *B. saltans* orthologs for many LAPs excludes association with parasitism, although we cannot exclude extreme divergence for lack of detection outside the kinetoplastids. Significantly, we recognize major differences between proteins comprising the lamina [8,9], kinetochores [106], mRNA processing [25,157] and export machinery [145,155,156] in trypanosomes and other lineages, including many lineage-specific components [8,9,106,145,155,156]. Interactions of multiple LAPs with the nuclear basket suggest a more complex structure than previously considered, with the potential that LAP71 and LAP102 mediate chromatin interactions. Finally, identification of architectural similarities between LAP333 and LAP92 and animal/fungal proteins was only enabled with recent advances in AI-mediated structure prediction and serves as a caution against an over-assumption of novelty based on sequence data alone.

## Supplementary Material

Supp Fig 13.jpg

Supp Fig 22.jpg

Supp Fig 26.jpg

Supp Fig 11.jpg

Supp Fig 18.jpg

Supp Fig 02.jpg

Supp Fig 07.jpg

Supp Fig 12.png

Supp Fig 21.docx

Supp Fig 25.jpg

Supp Fig 05.jpg

Supp Fig 08.jpg

Supp Fig 23.jpg

Supp Fig 03.jpg

Supp Fig 20.docx

Supp Fig 09.jpg

Butterfield et al Supplementary Tables.xlsx

Supp Fig 01.jpg

Supp Fig 27.png

Supp Fig 06.jpg

Supp Fig 04.jpg

Supp Fig 19.png

Supp Fig 15.jpg

Supp Fig 24.jpg

Supp Fig 10.jpg

Supp Fig 16.jpg

Supp Fig 14.jpg

Supp Fig 17.jpg

## Data Availability

LAP accessions and sequences are available in Supplementary Tables S5 and S6. Generated models are available in ModelArchive (modelarchive.org) with the accession codes: ma-idy3n (LAP71), ma-2or17 (LAP92), ma-cit5c (EgLAP59), ma-a7kc8, ma-hr1rc, ma-x4kzz, ma-rca1e (DeepMind monomer models F1, F2A, F2B and F3 respectively), ma-i35ix, ma-p3ykg, ma-1anz9 (ColabFold monomer models N-terminal, middle and C-terminal respectively), ma-jus11 (DeepMind multimer), ma-j1lld (ColabFold multimer), ma-8116p, ma-zoydh, ma-8nwhc and ma-ru9va (LAP333 and LAP59 complex models F1, F2A, F2B and F3 respectively). Scripts are available in GitHub, at https://github.com/erin-r-butterfield/LAPs. Proteomics data are available in Zenodo, at https://zenodo.org/record/8273805. The transcriptome data, alignments and phylogenetic trees are available in Zenodo, at https://zenodo.org/record/8355677. The transcriptome datasets were derived from sources in the public domain (European Nucleotide Archive at https://www.ebi.ac.uk/ena/browser/home, accessions: PRJEB3146 (*B. saltans*) [75,76] and PRJNA414522 (sample SAMN07793202) (*P. confusum*) [79,80]. Additional sequencing data analysed are present in the public domain (European Nucleotide Archive at https://www.ebi.ac.uk/ena/browser/home, accessions: PRJEB36170 (*A. deanei* genome)[73,74] and PRJNA680236 (*E. monterogeii* RNA-seq) [77,78]. Precomputed AlphaFold models are available in the AlphaFold Protein Structure Database (https://alphafold.ebi.ac.uk/), accessions: Q57X92 (LAP59), Q583W2 (LAP73), Q581B5 (LAP102), Q585F7 (LAP173), Q9NXE4 (HsnSMase3), P39685 (ScPom152), Q8TEM1 (HsNup210), Q12445 (ScPom34), Q4QJ68 (LmLAP59) and LAP59 orthologs (accessions listed in Supplementary Table S5).
