# Supplementary figures and images for: A lineage-specific protein network at the trypanosome nuclear envelope
Source: Nucleus. 2024 Apr 11;15(1):2310452. doi: 10.1080/19491034.2024.2310452 (PMC11018031; doi:10.1080/19491034.2024.2310452)

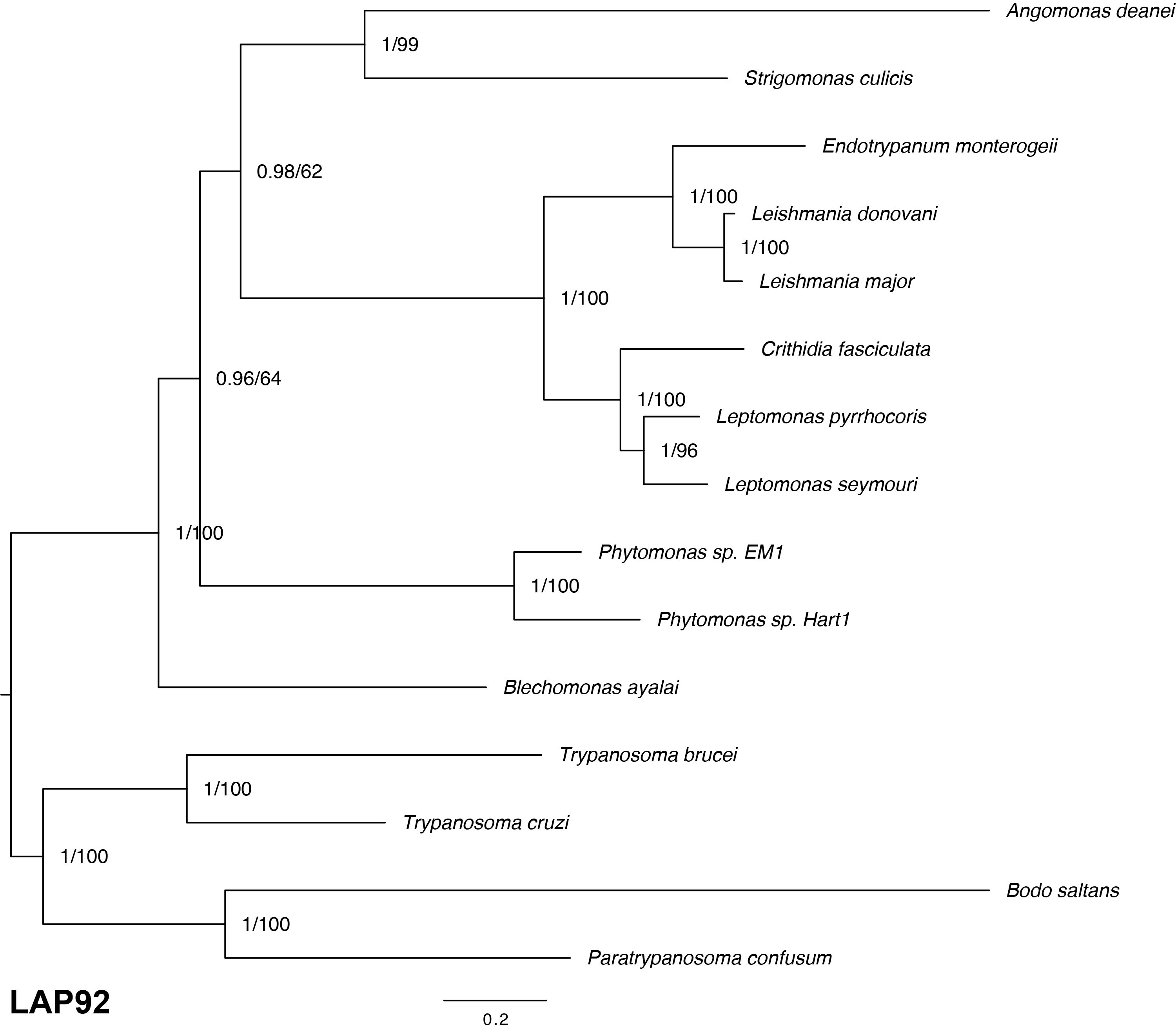

Supplement: Supp Fig 13.jpg [file KNCL_A_2310452_SM3311.jpg]

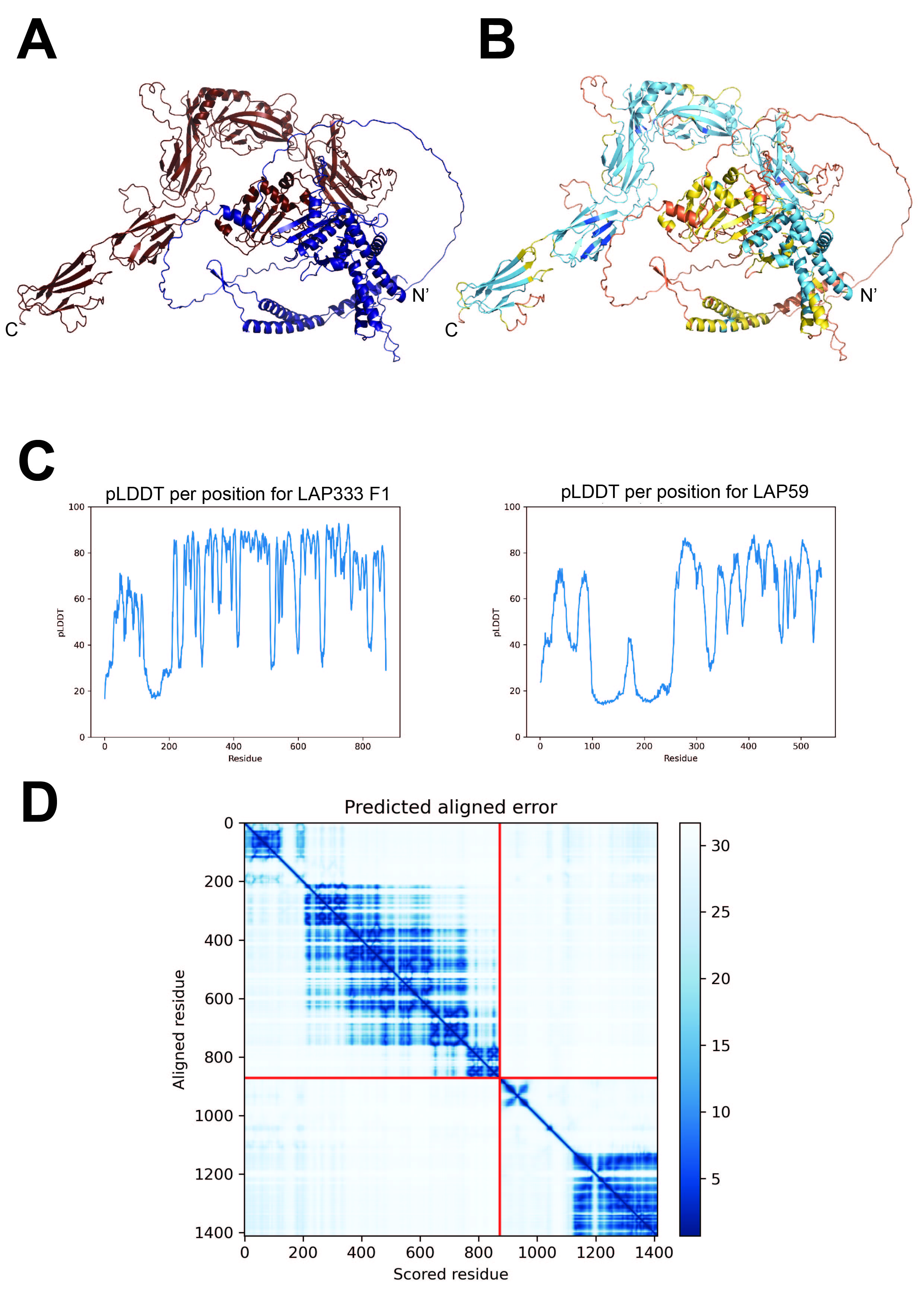

Supplement: Supp Fig 22.jpg [file KNCL_A_2310452_SM3310.jpg]

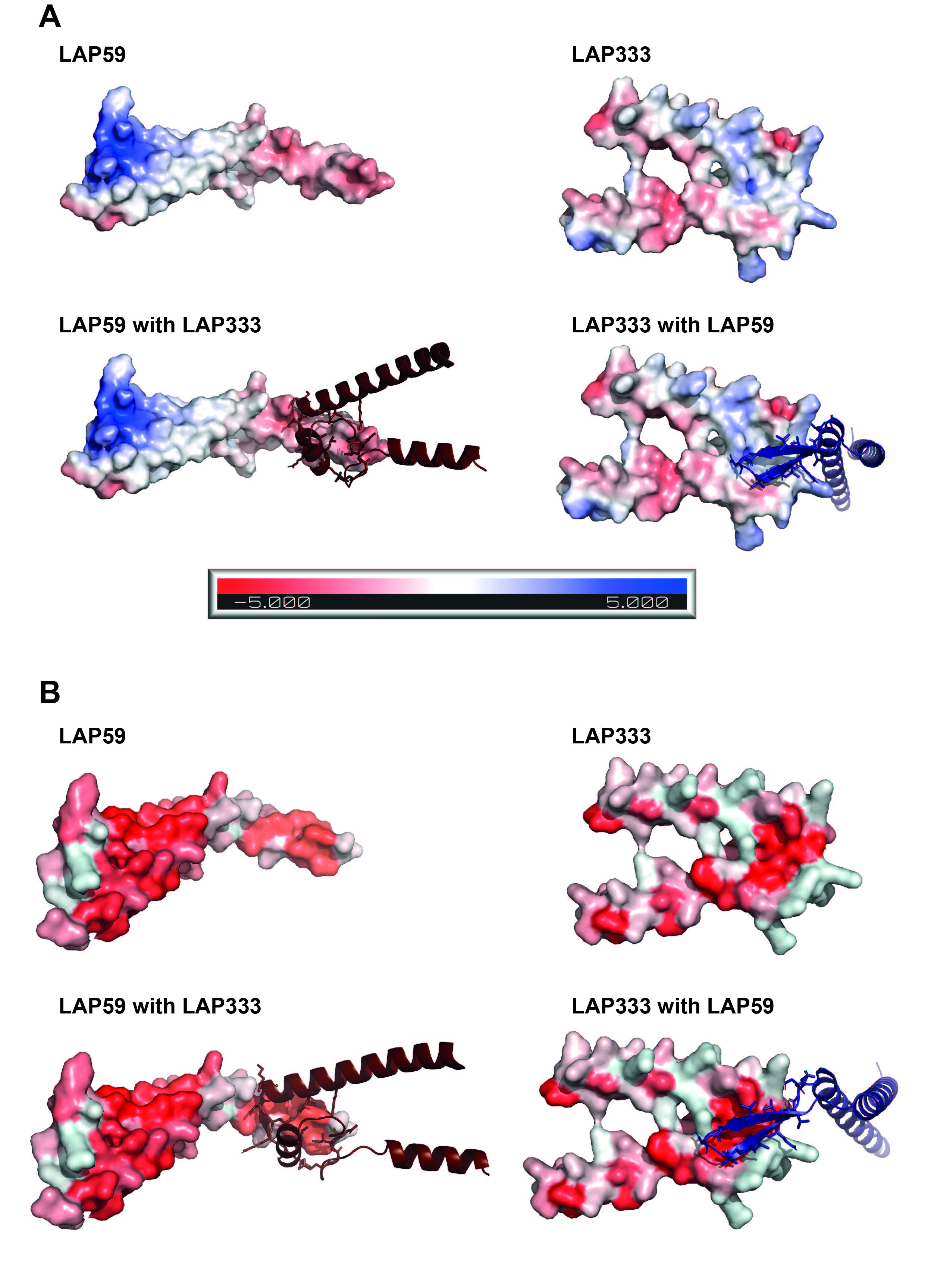

Supplement: Supp Fig 26.jpg [file KNCL_A_2310452_SM3309.jpg]

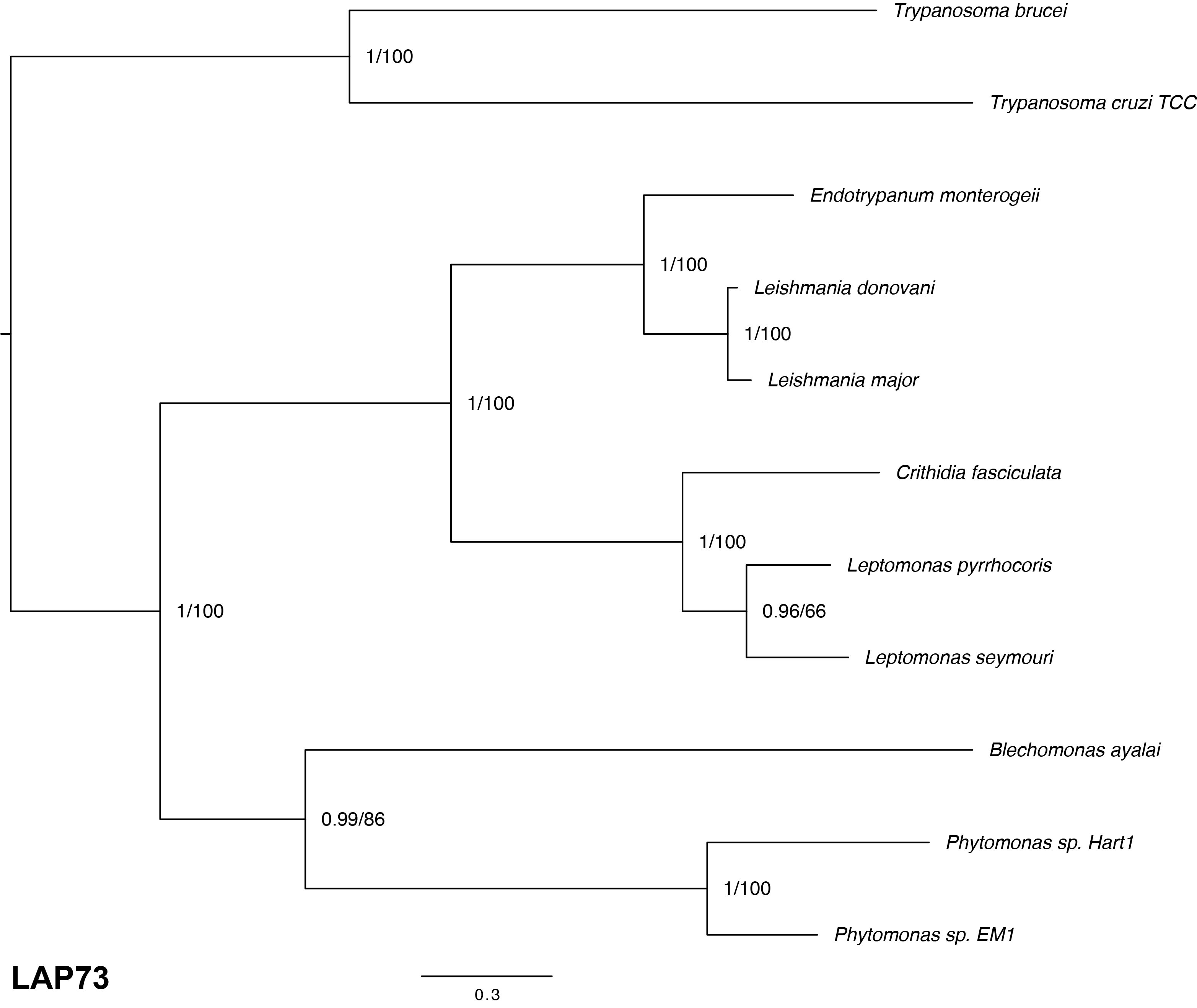

Supplement: Supp Fig 11.jpg [file KNCL_A_2310452_SM3308.jpg]

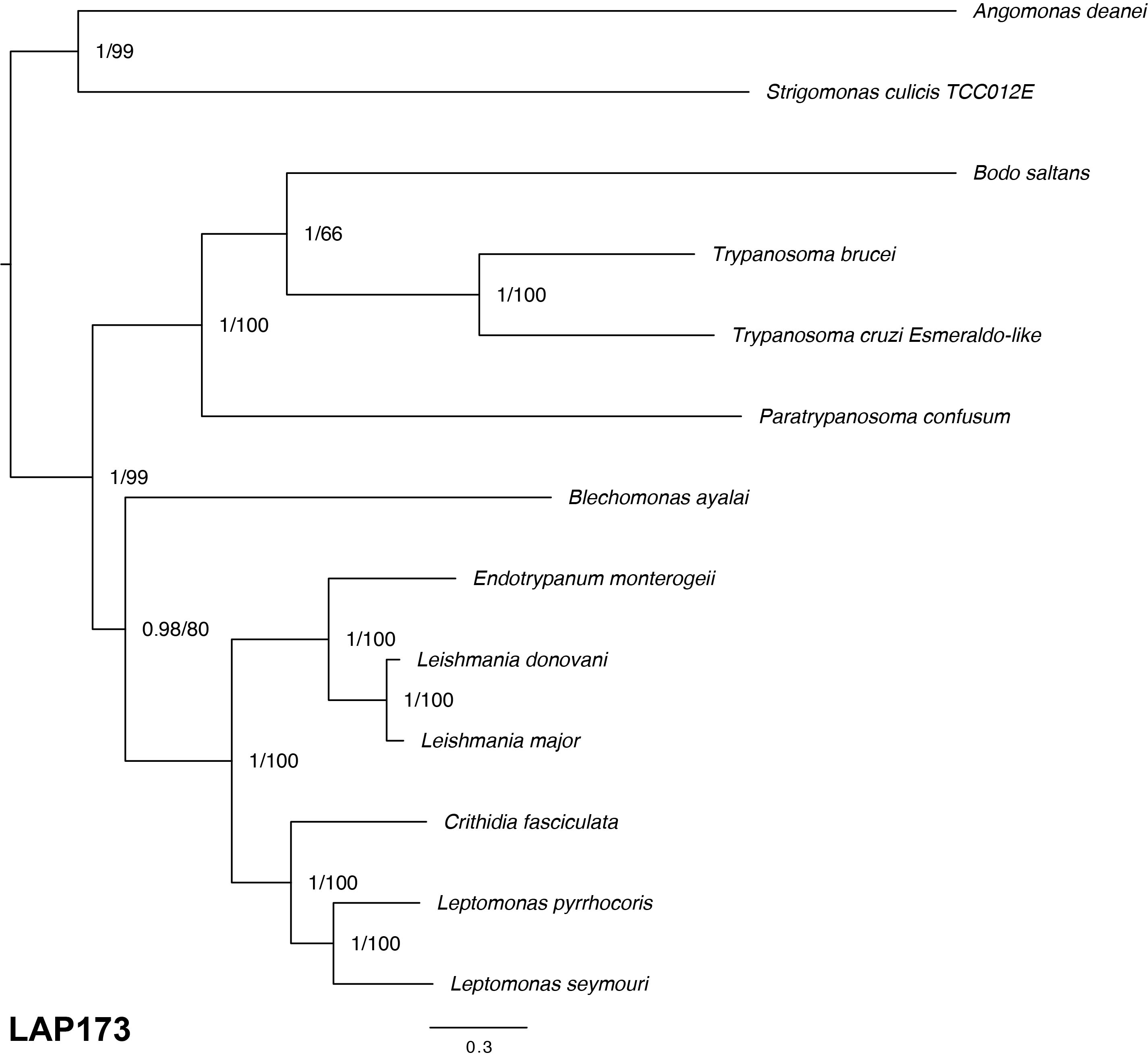

Supplement: Supp Fig 18.jpg [file KNCL_A_2310452_SM3307.jpg]

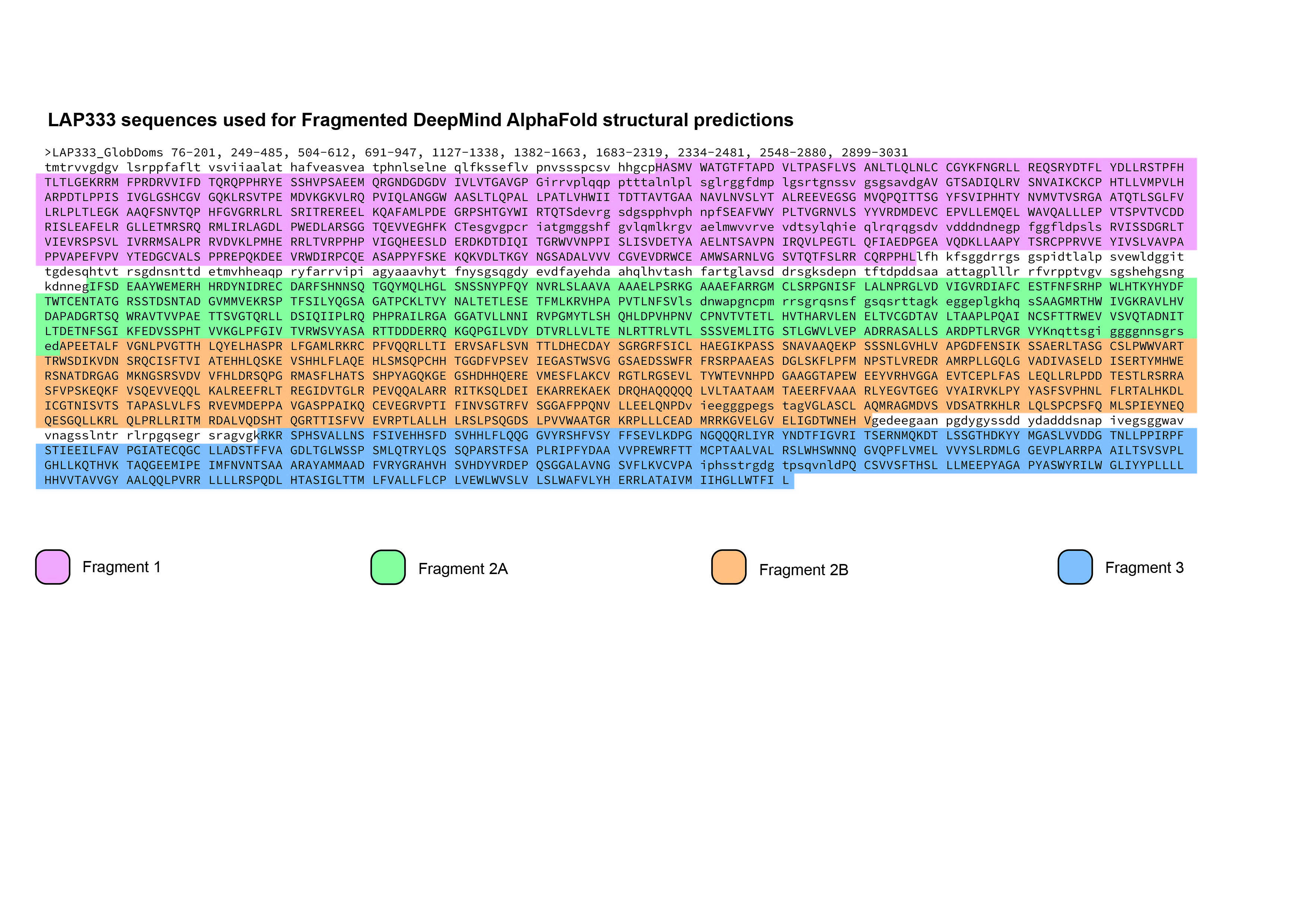

Supplement: Supp Fig 02.jpg [file KNCL_A_2310452_SM3306.jpg]

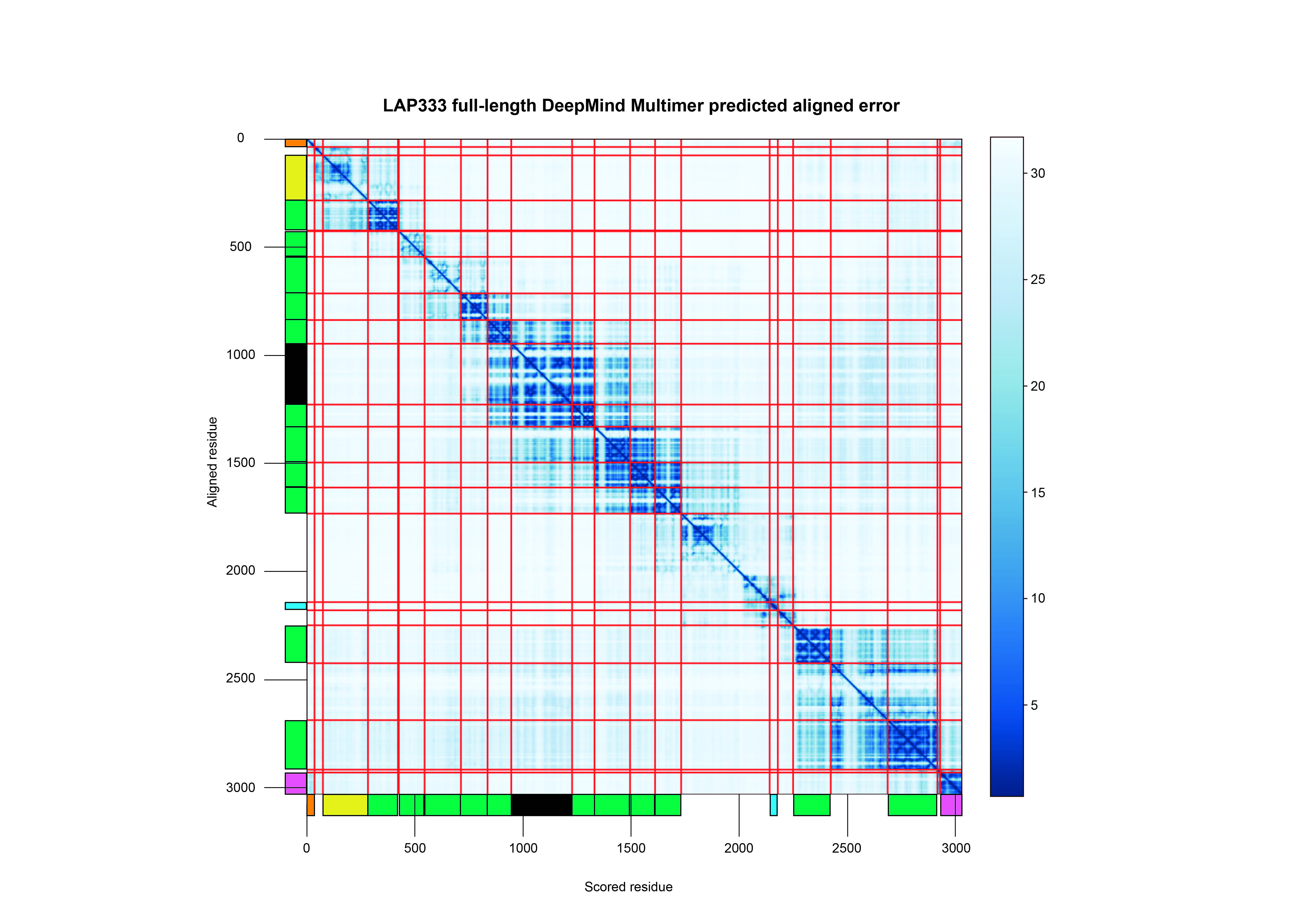

Supplement: Supp Fig 07.jpg [file KNCL_A_2310452_SM3305.jpg]

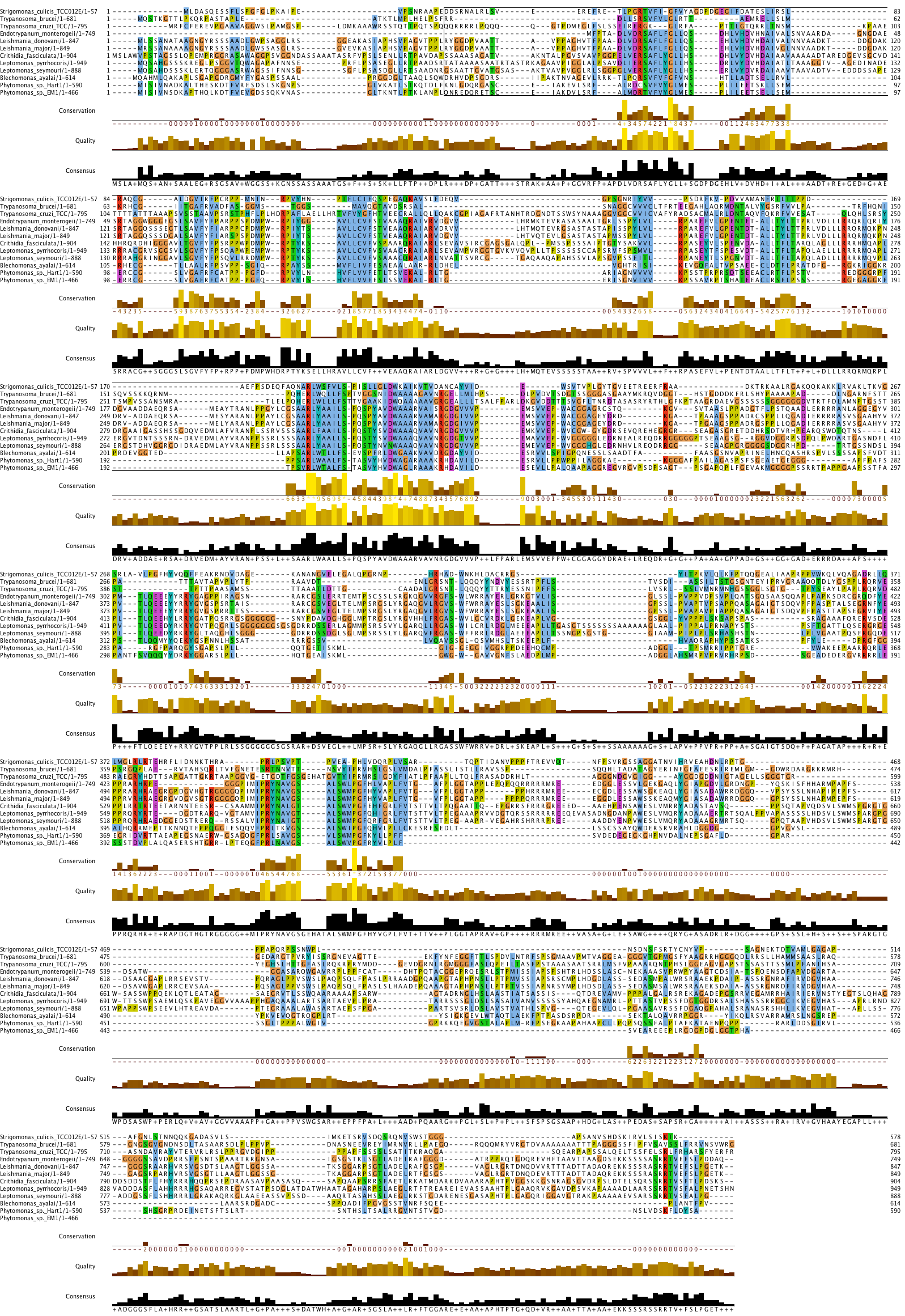

Supplement: Supp Fig 12.png [file KNCL_A_2310452_SM3304.png]

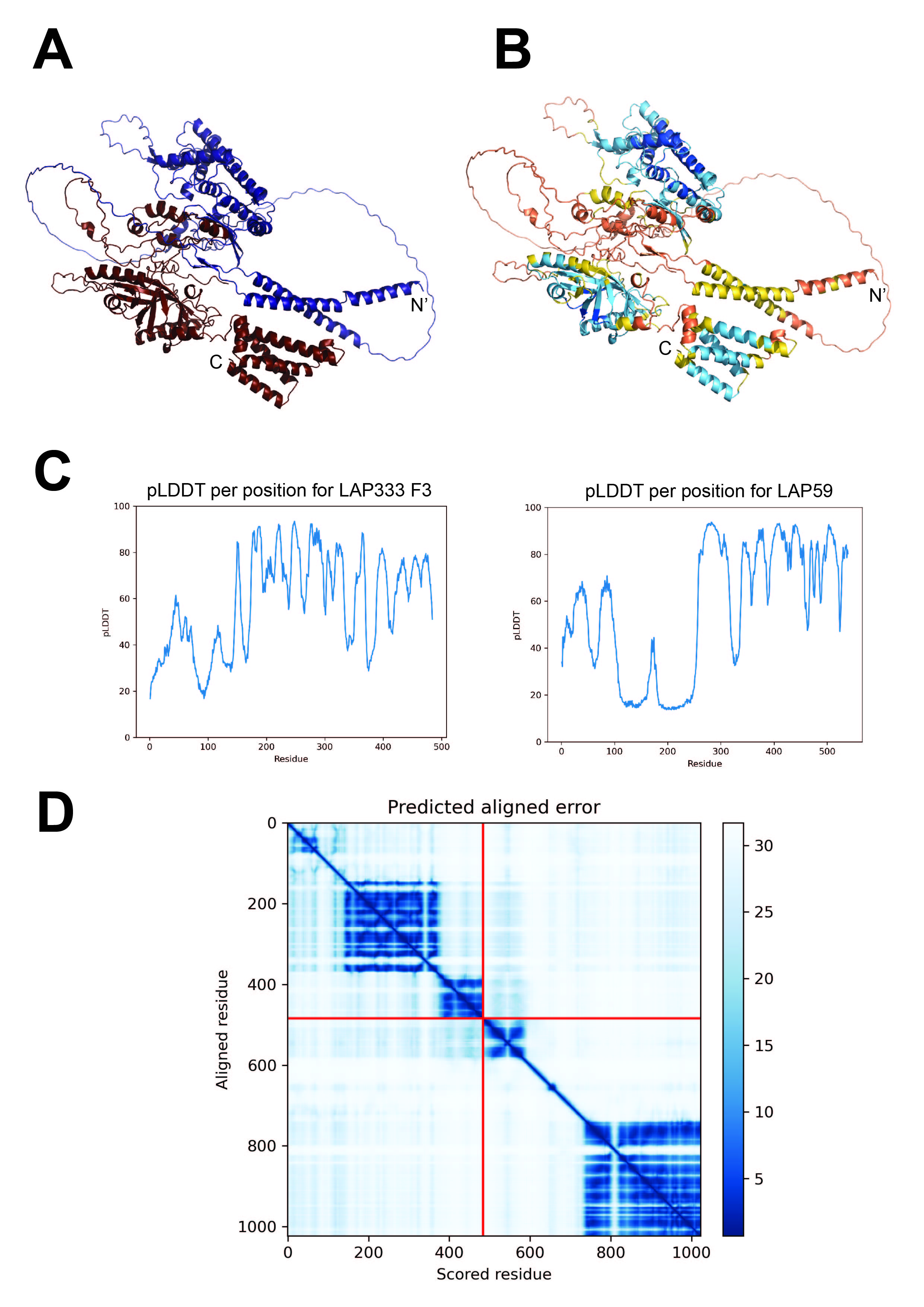

Supplement: Supp Fig 25.jpg [file KNCL_A_2310452_SM3302.jpg]

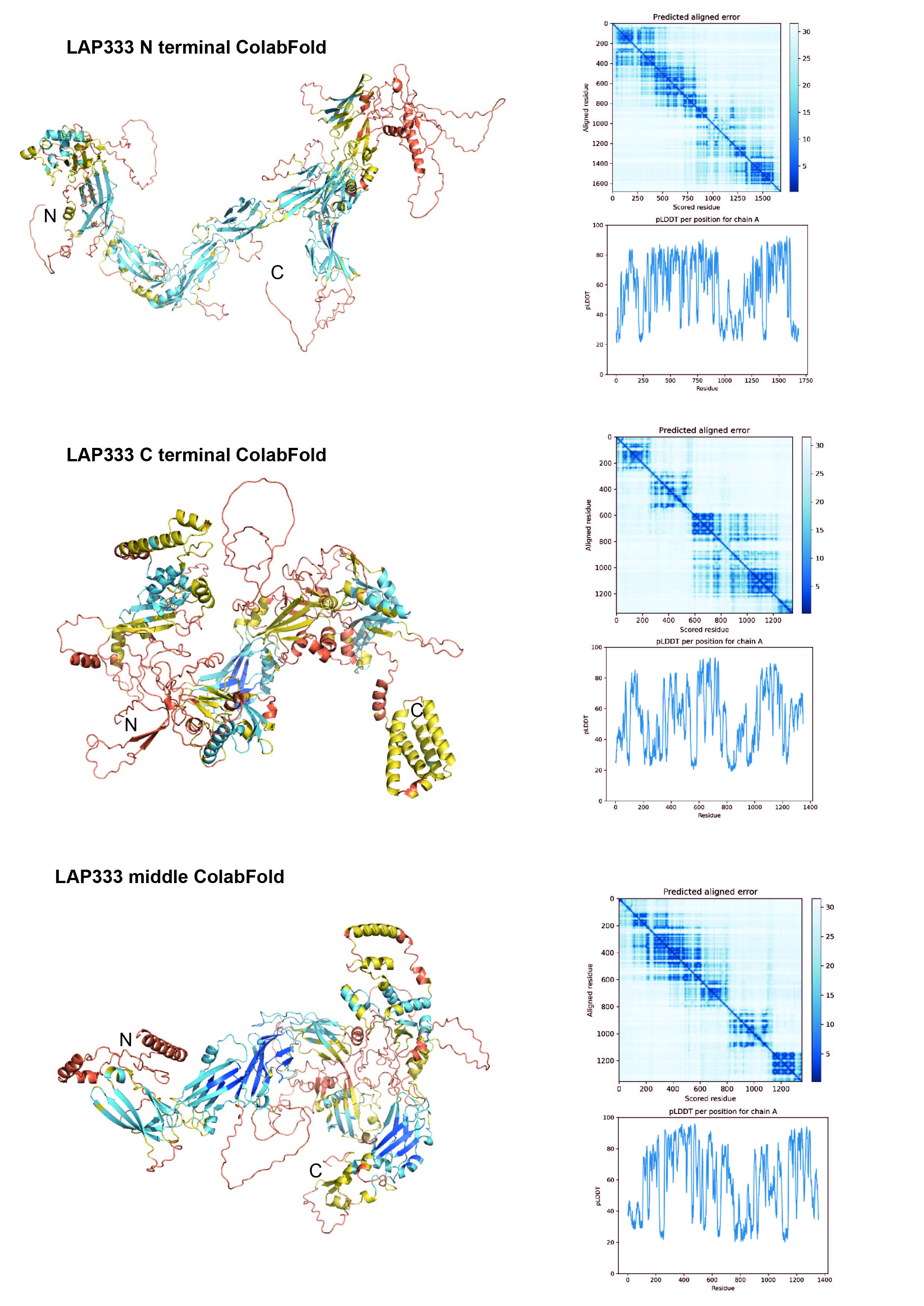

Supplement: Supp Fig 05.jpg [file KNCL_A_2310452_SM3301.jpg]

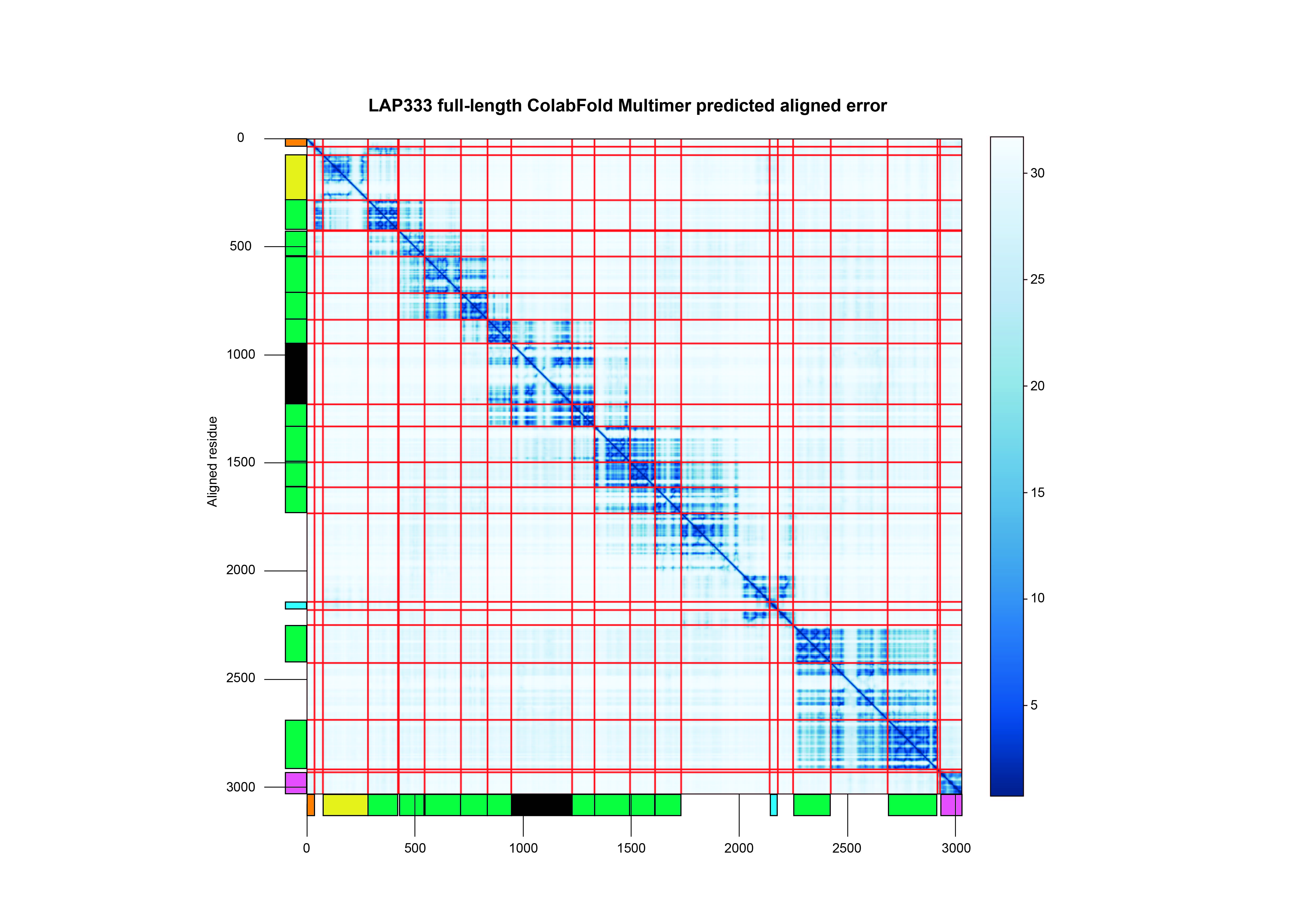

Supplement: Supp Fig 08.jpg [file KNCL_A_2310452_SM3300.jpg]

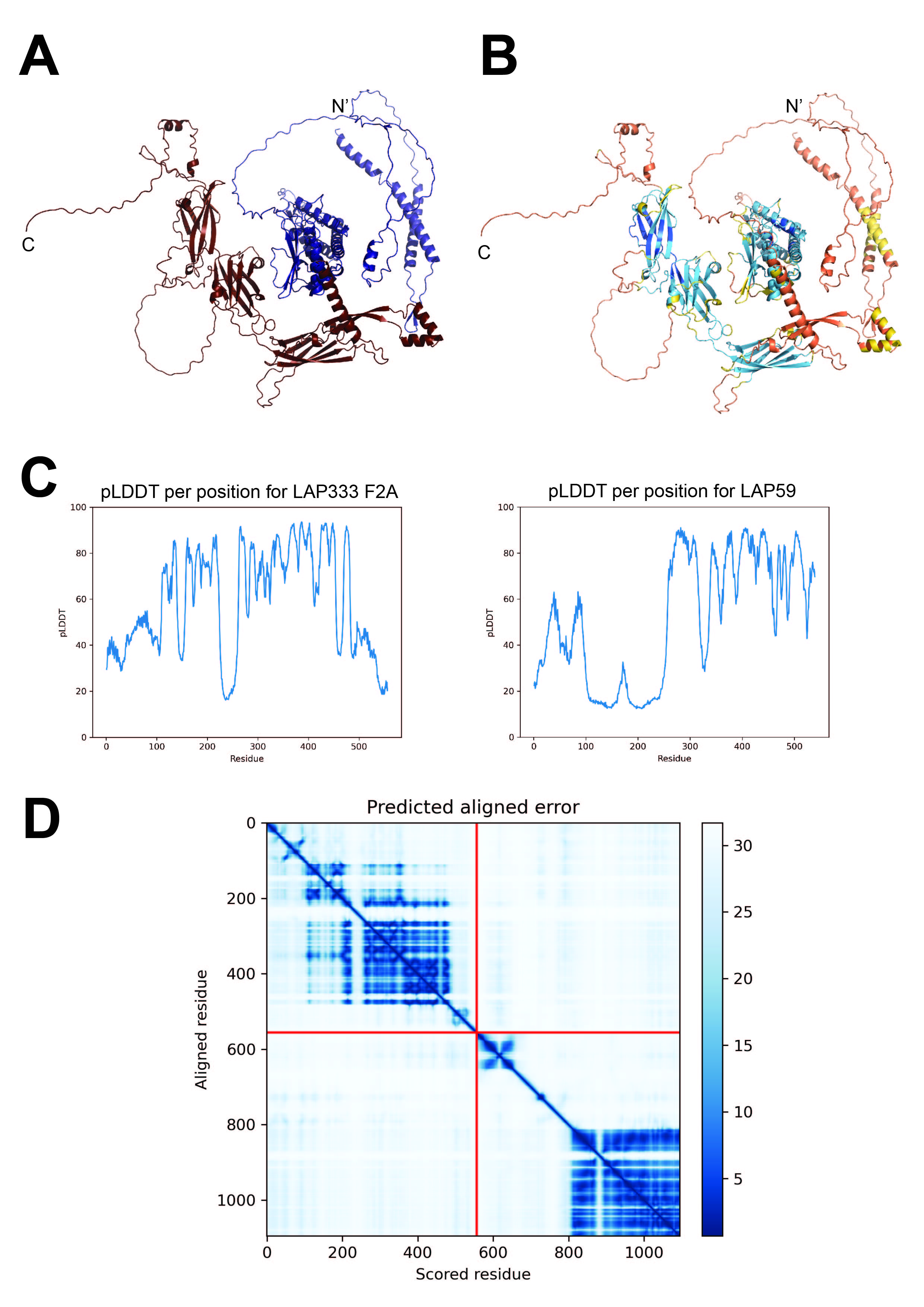

Supplement: Supp Fig 23.jpg [file KNCL_A_2310452_SM3299.jpg]

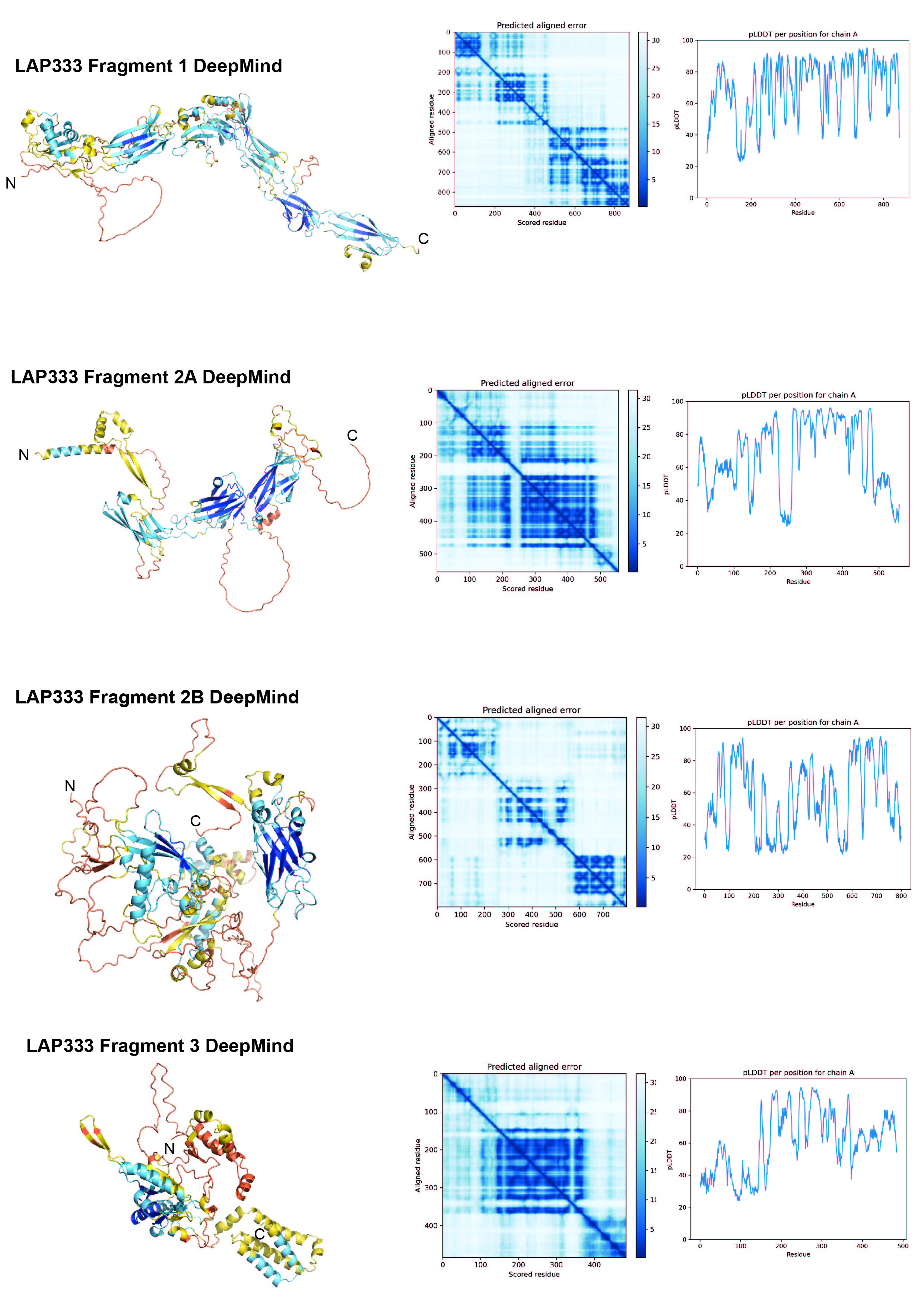

Supplement: Supp Fig 03.jpg [file KNCL_A_2310452_SM3298.jpg]

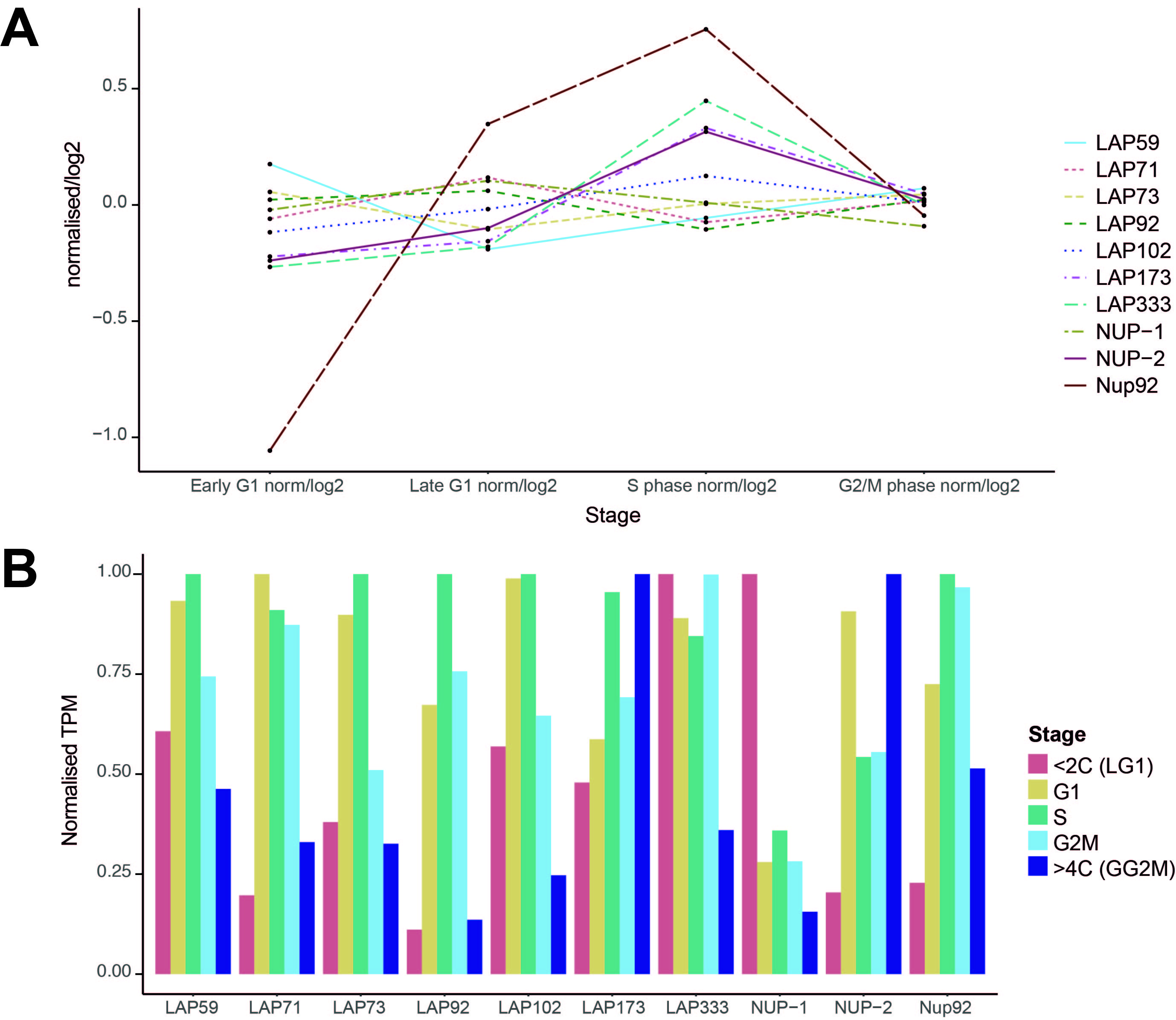

Supplement: Supp Fig 09.jpg [file KNCL_A_2310452_SM3296.jpg]

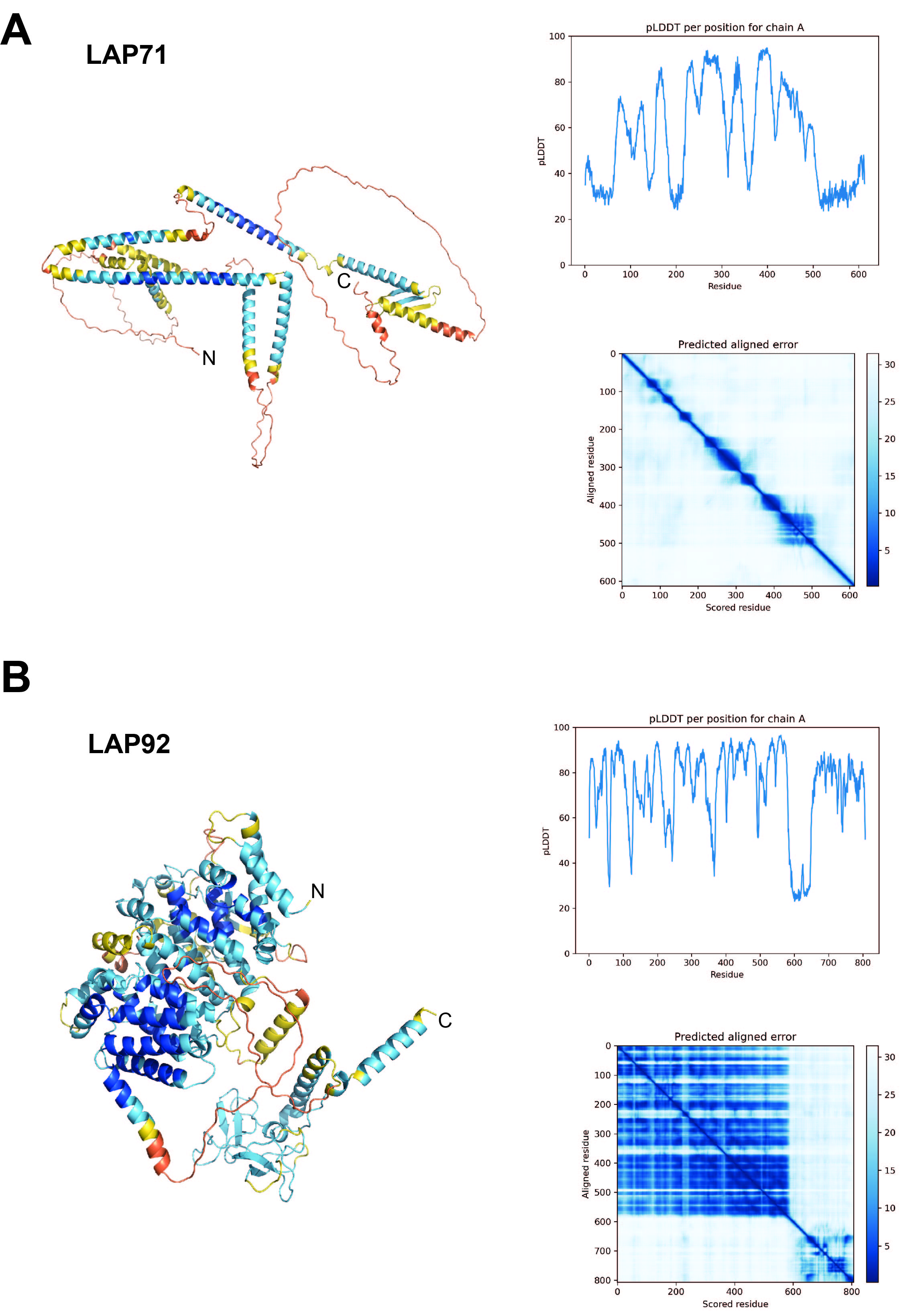

Supplement: Supp Fig 01.jpg [file KNCL_A_2310452_SM3294.jpg]

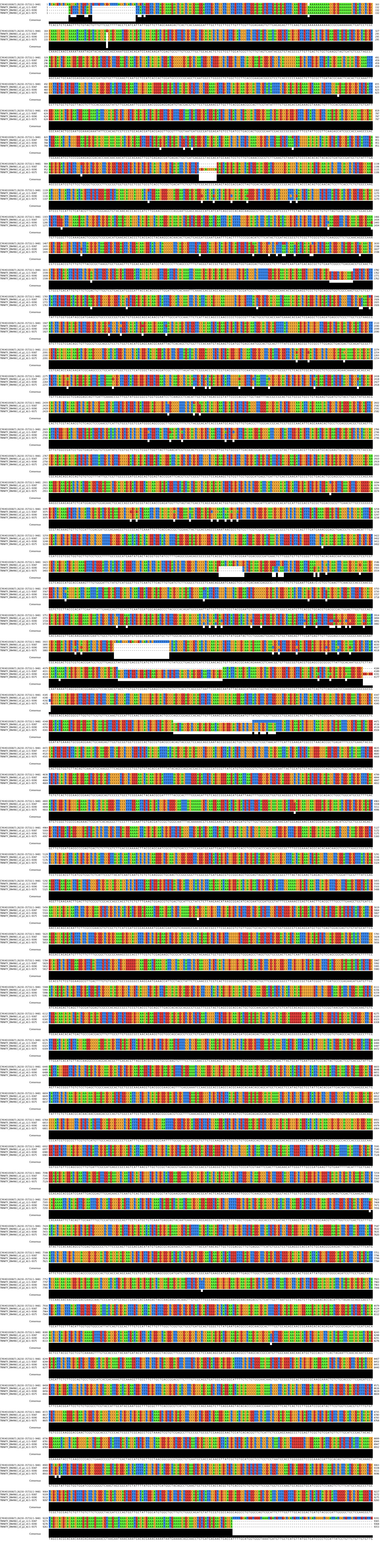

Supplement: Supp Fig 27.png [file KNCL_A_2310452_SM3293.png]

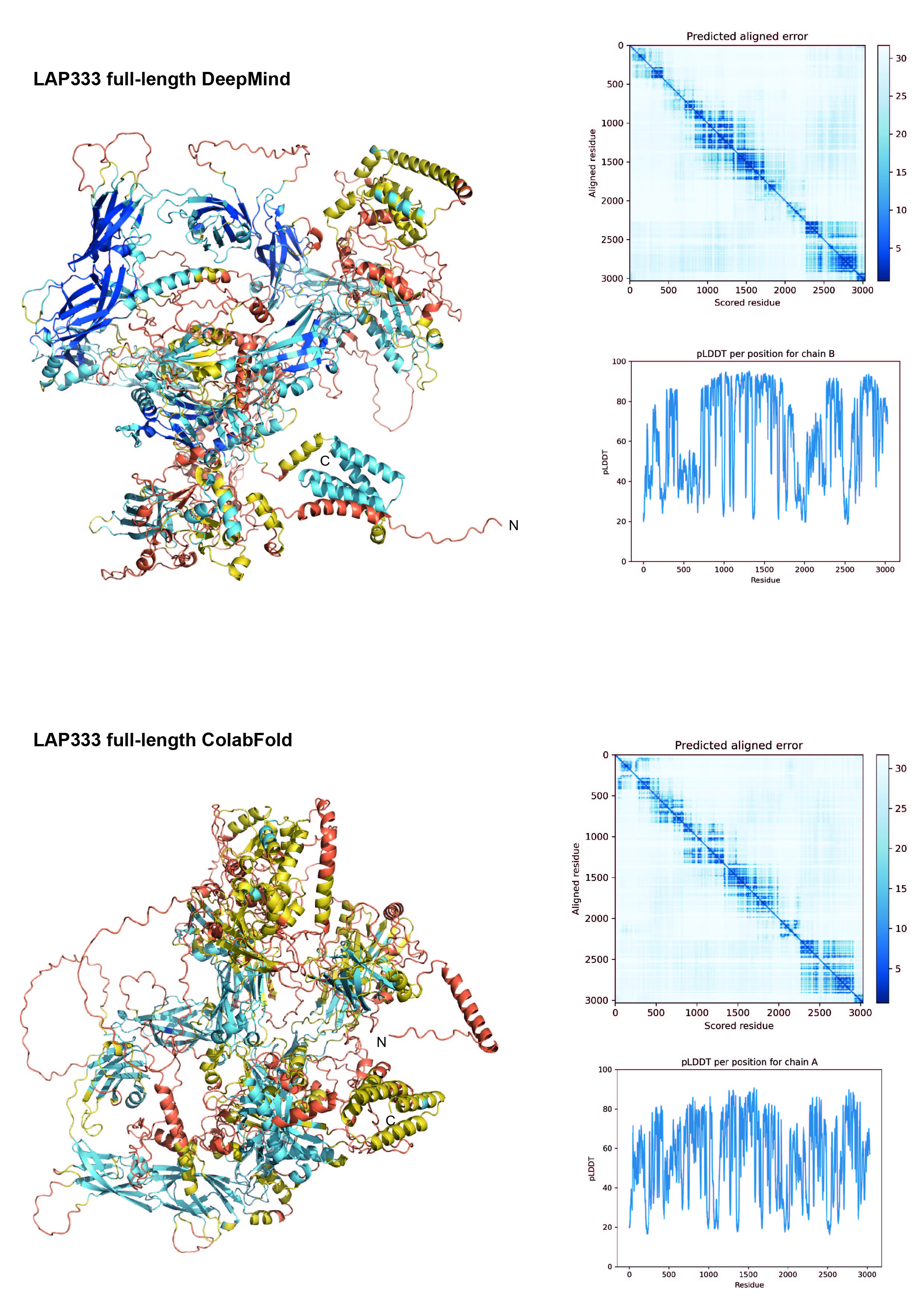

Supplement: Supp Fig 06.jpg [file KNCL_A_2310452_SM3292.jpg]

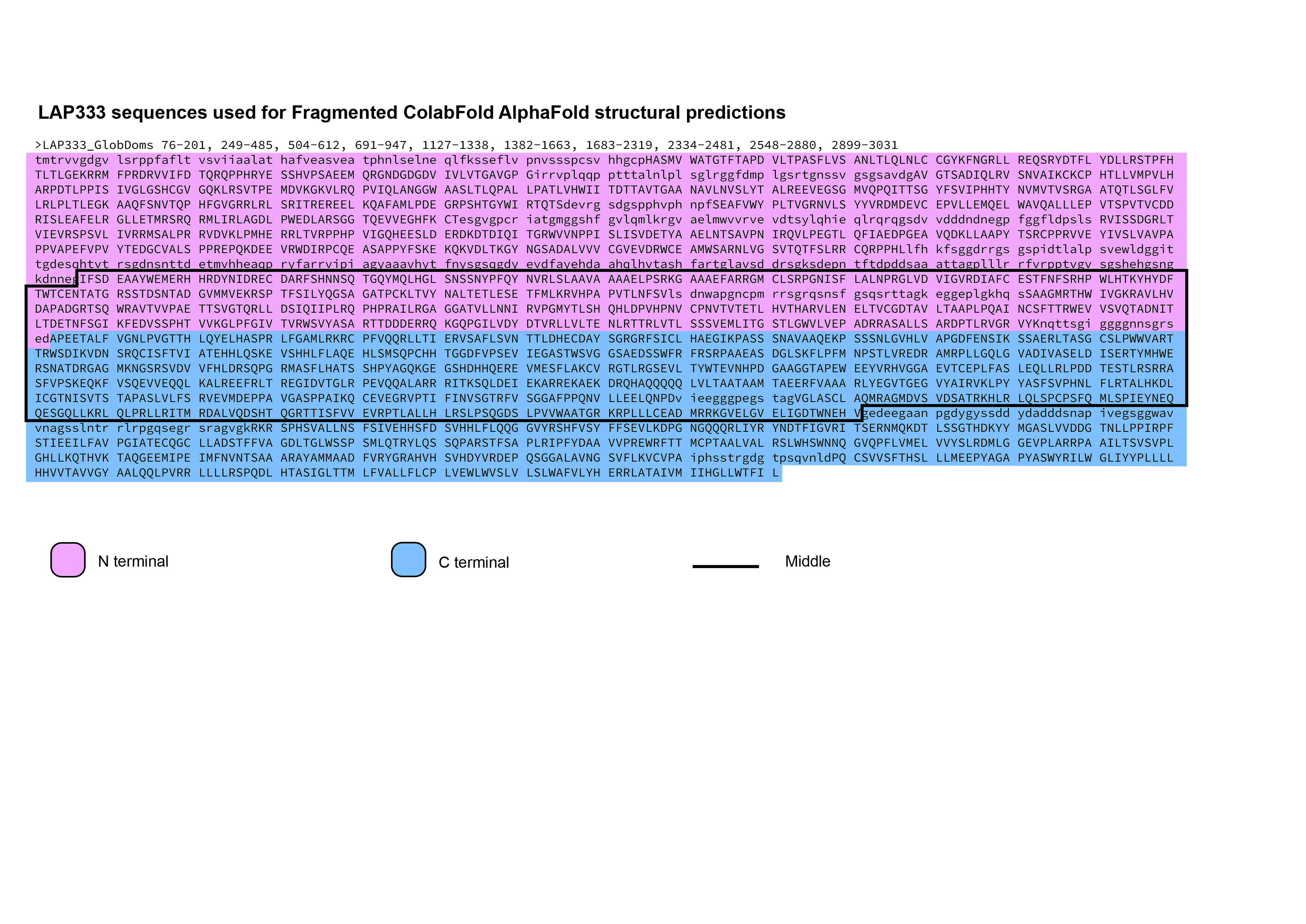

Supplement: Supp Fig 04.jpg [file KNCL_A_2310452_SM3291.jpg]

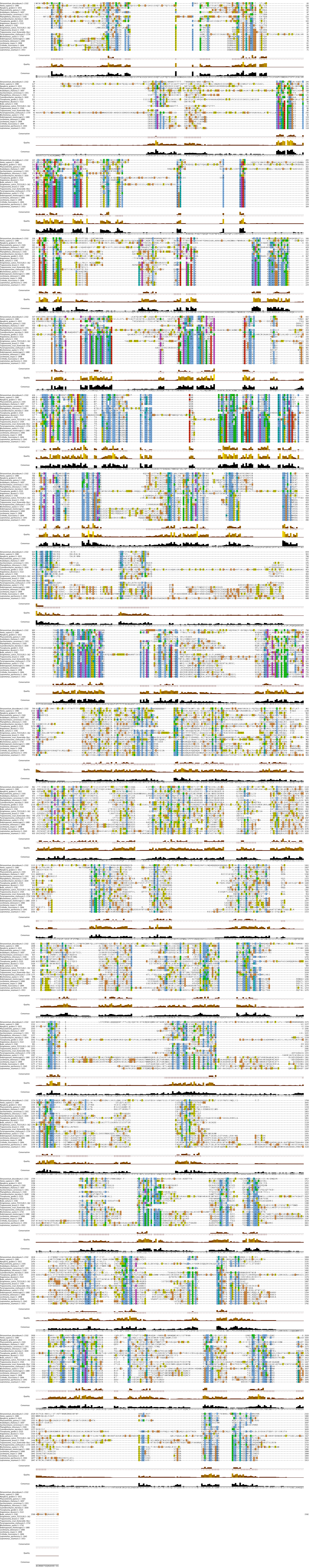

Supplement: Supp Fig 19.png [file KNCL_A_2310452_SM3290.png]

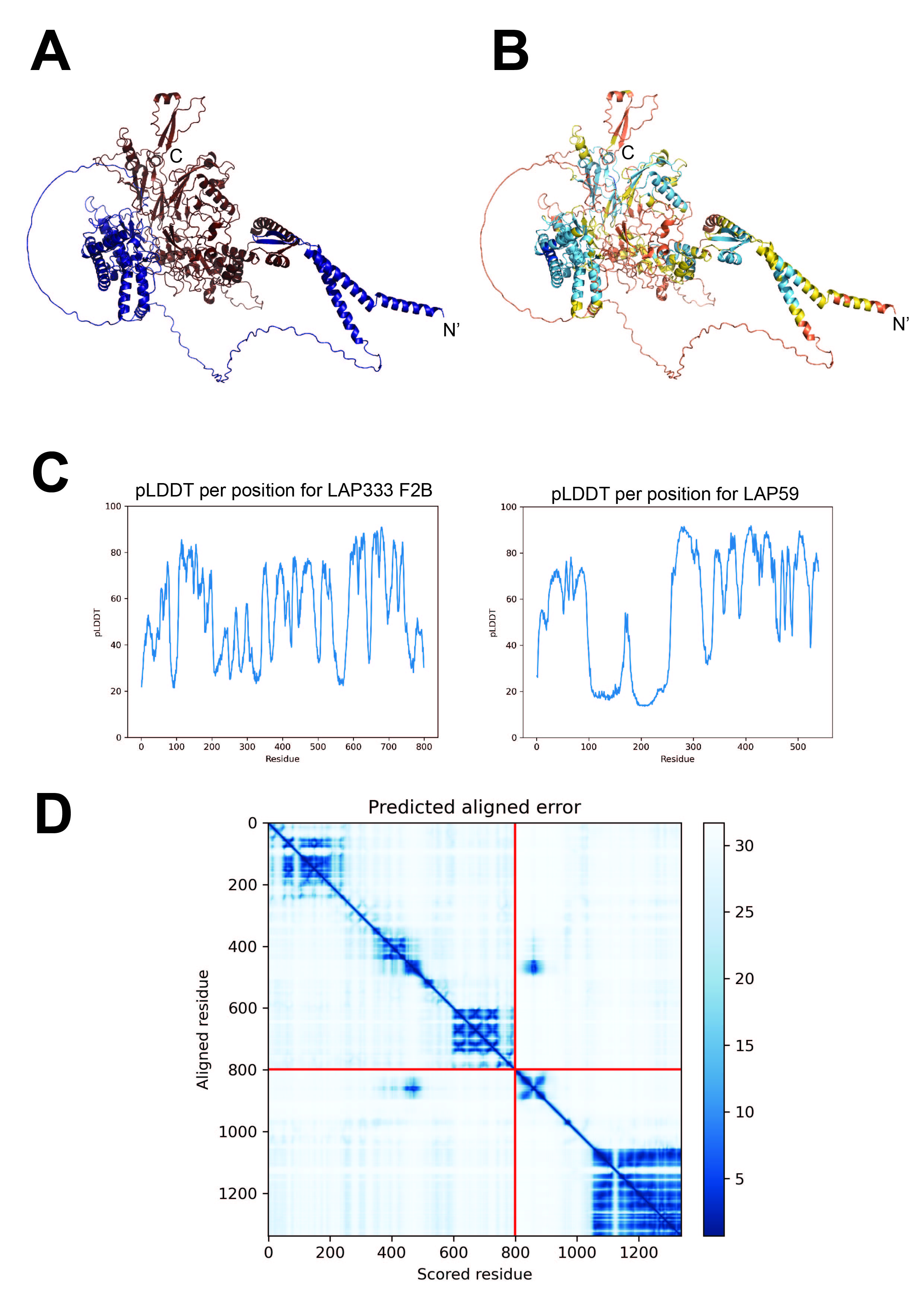

Supplement: Supp Fig 24.jpg [file KNCL_A_2310452_SM3288.jpg]

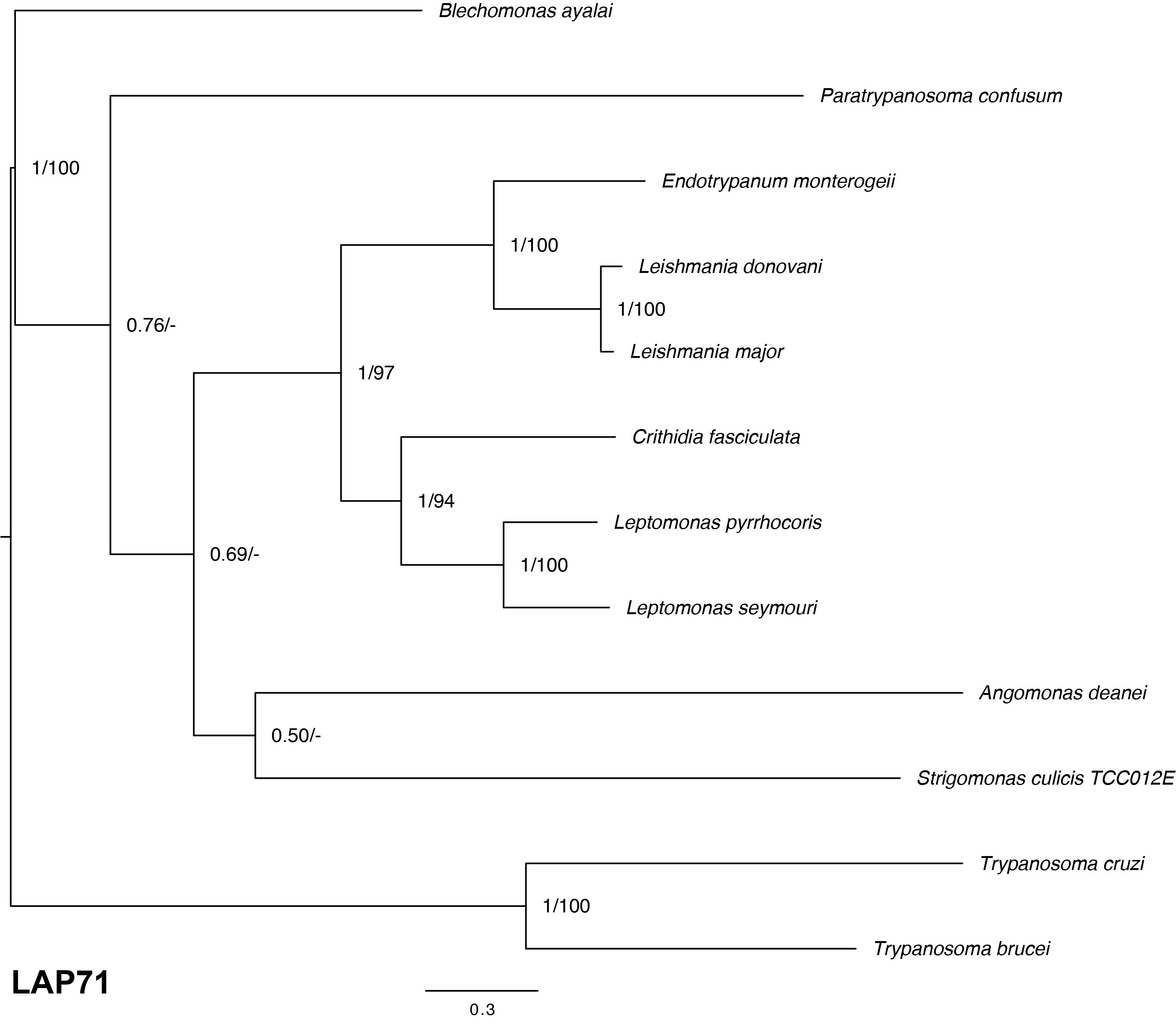

Supplement: Supp Fig 10.jpg [file KNCL_A_2310452_SM3287.jpg]

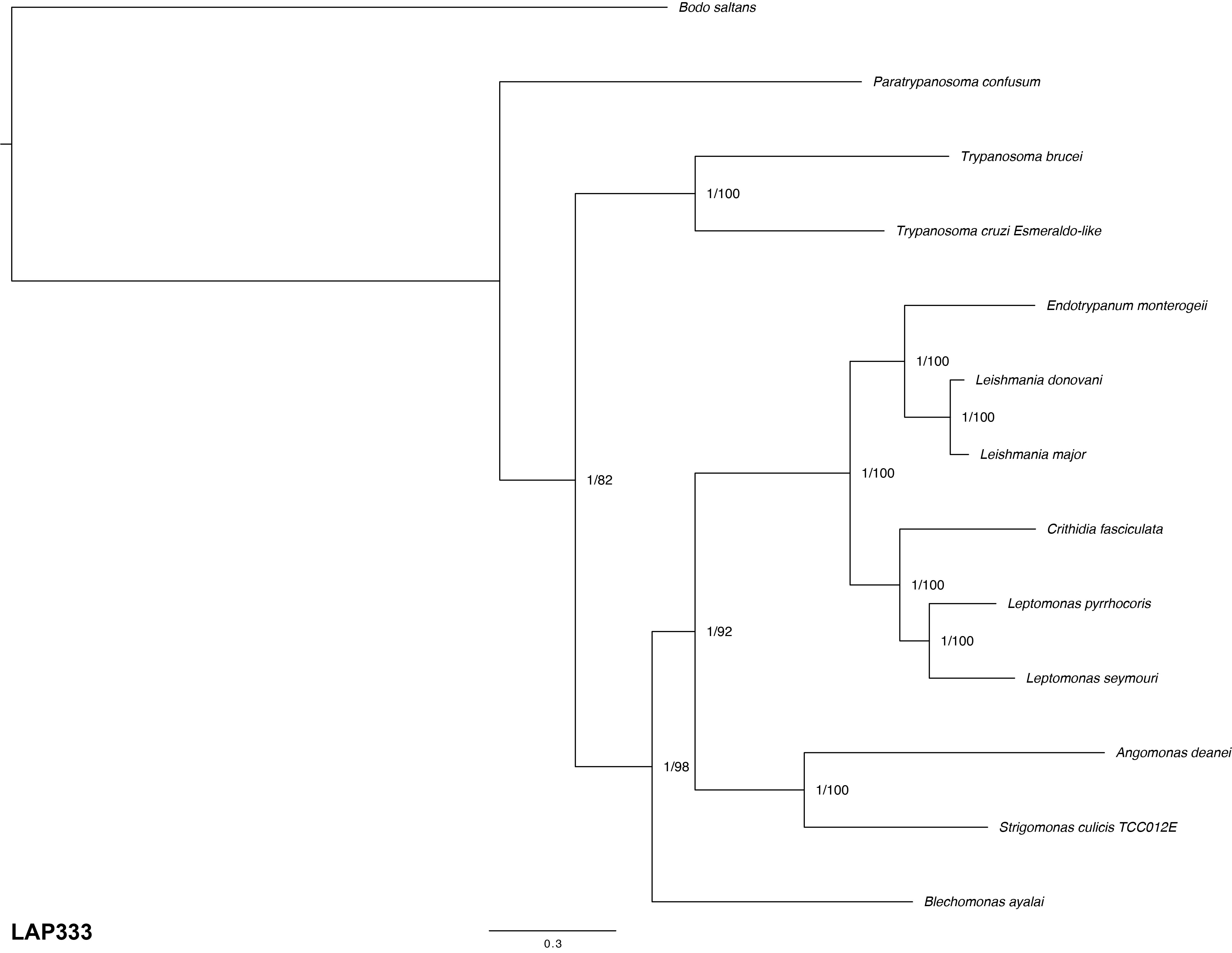

Supplement: Supp Fig 16.jpg [file KNCL_A_2310452_SM3286.jpg]

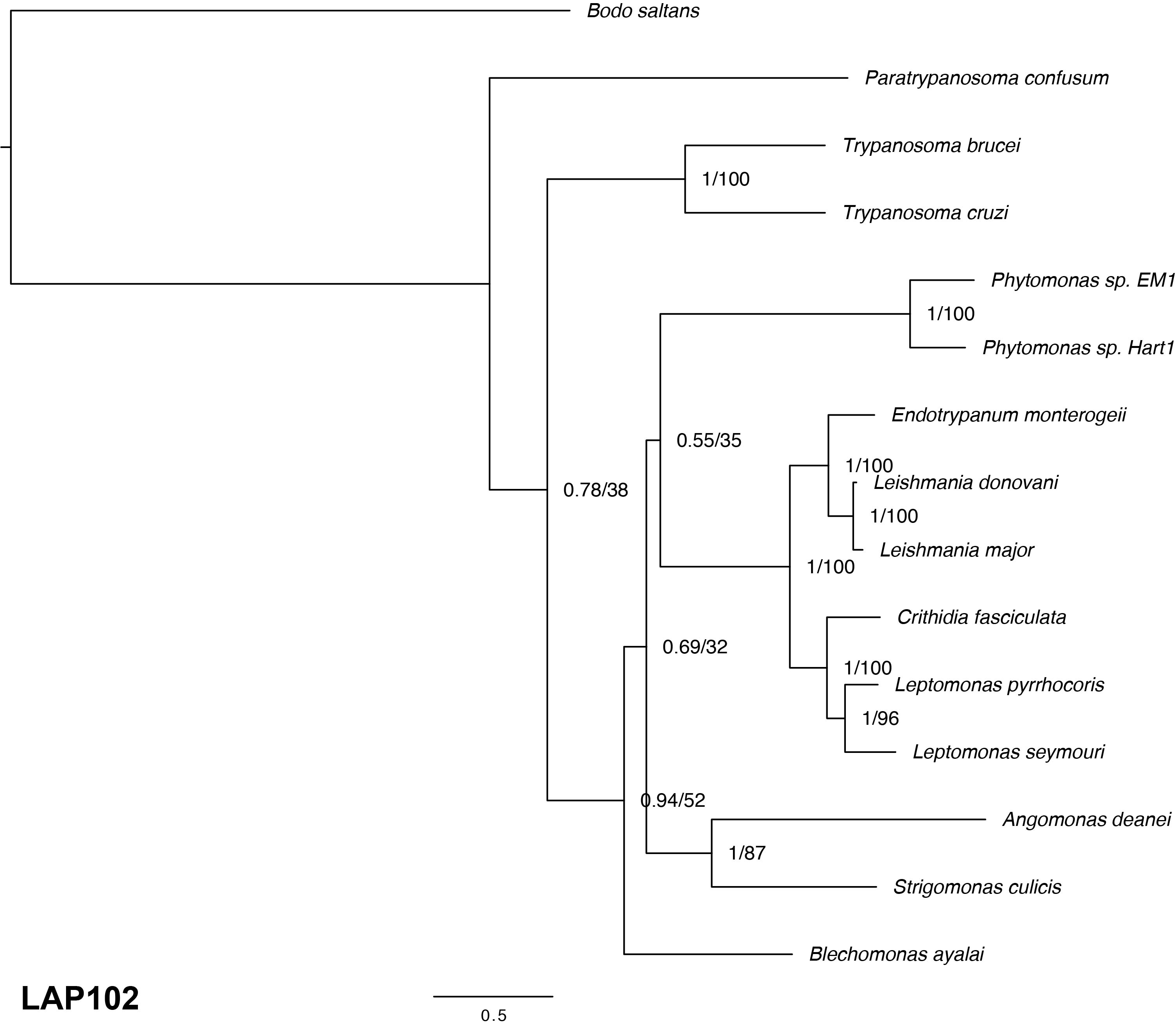

Supplement: Supp Fig 14.jpg [file KNCL_A_2310452_SM3285.jpg]

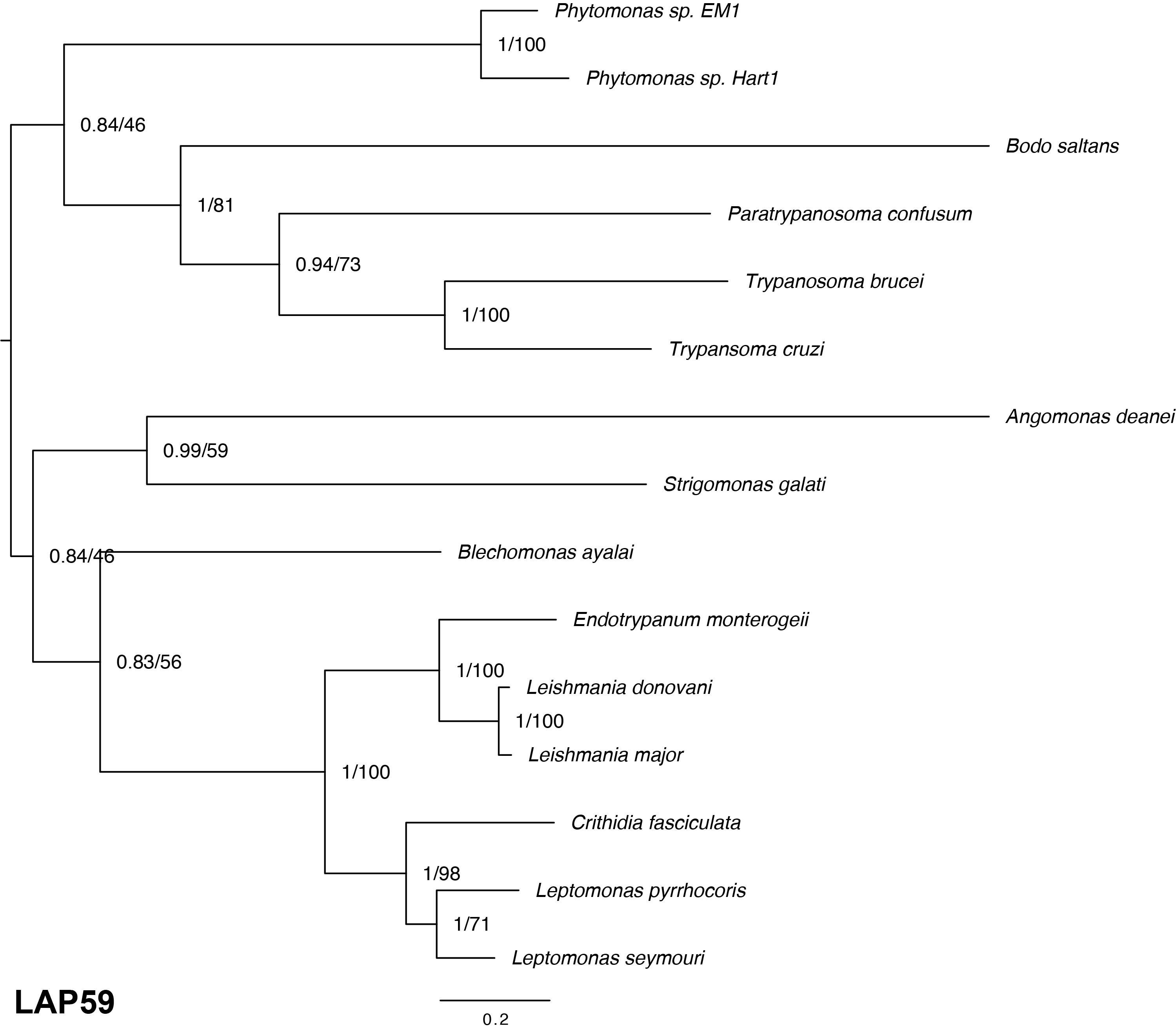

Supplement: Supp Fig 17.jpg [file KNCL_A_2310452_SM3284.jpg]
