# Supplementary material for: A lineage-specific protein network at the trypanosome nuclear envelope
Source: Nucleus. 2024 Apr 11;15(1):2310452. doi: 10.1080/19491034.2024.2310452 (PMC11018031; doi:10.1080/19491034.2024.2310452)
Supplement: Supp Fig 21.docx [file KNCL_A_2310452_SM3303.docx]

| **Protein** | **Stage** | **Mean** | **Median** | **Standard deviation** | **No. of Cells** |
| --- | --- | --- | --- | --- | --- |
| LAP71 | Interphase | 0.62361905 | 0.632 | 0.15467142 | 21 |
|  | G2 phase | 0.598 | 0.599 | 0.09999429 | 8 |
|  | Post mitosis | 0.69428571 | 0.7205 | 0.11364143 | 7* |
| LAP73 | Interphase | 0.58326316 | 0.574 | 0.12243086 | 19 |
|  | G2 phase | 0.4452 | 0.416 | 0.217655 | 5 |
|  | Post mitosis | 0.37925 | 0.3905 | 0.15454334 | 4* |
| LAP102 | Interphase | 0.72478571 | 0.757 | 0.10715638 | 14 |
|  | G2 phase | 0.70191667 | 0.7105 | 0.14825314 | 12 |
|  | Post mitosis | 0.33342857 | 0.396 | 0.26916358 | 7* |
