# Supplementary material for: A lineage-specific protein network at the trypanosome nuclear envelope
Source: Nucleus. 2024 Apr 11;15(1):2310452. doi: 10.1080/19491034.2024.2310452 (PMC11018031; doi:10.1080/19491034.2024.2310452)
Supplement: Supp Fig 20.docx [file KNCL_A_2310452_SM3297.docx]

| **Protein** | **Stage** | **Mean** | **Median** | **Standard deviation** | **No. of Cells** |
| --- | --- | --- | --- | --- | --- |
| LAP71 | Interphase | 0.57121739 | 0.591 | 0.11651569 | 23 |
|  | G2 phase | 0.53928571 | 0.513 | 0.14304395 | 7 |
|  | Post mitosis | 0.58857143 | 0.6335 | 0.19656736 | 7* |
| LAP73 | Interphase | 0.07273077 | 0.111 | 0.21174939 | 26 |
|  | G2 phase | 0.09366667 | 0.1395 | 0.23809446 | 12 |
|  | Post mitosis | 0.00666667 | -0.0375 | 0.28204276 | 9* |
| LAP102 | Interphase | 0.60863636 | 0.613 | 0.14857609 | 11 |
|  | G2 phase | 0.6426 | 0.612 | 0.10713916 | 5 |
|  | Post mitosis | 0.57216667 | 0.5545 | 0.12126569 | 3* |
